# Modulation of mouse laryngeal inflammatory and immune cell responses by low and high doses of mainstream cigarette smoke

**DOI:** 10.1038/s41598-022-23359-7

**Published:** 2022-11-04

**Authors:** Meena Easwaran, Joshua D. Martinez, Juyong Brian Kim, Elizabeth Erickson-DiRenzo

**Affiliations:** 1 Division of Laryngology, Department of Otolaryngology-Head and Neck Surgery, Stanford University School of Medicine, Stanford, CA USA; 2 Department of Cardiovascular Medicine, Stanford University School of Medicine, Stanford, CA USA

**Keywords:** Gene expression profiling, Immunohistochemistry, Bioinformatics, Respiratory tract diseases, Experimental models of disease

## Abstract

Cigarette smoking is a major risk factor for laryngeal diseases. Despite well-documented cigarette smoke (CS) induced laryngeal histopathological changes, the underlying immunopathological mechanisms remain largely unexplored. The goal of this study was to evaluate inflammatory and immune cell responses in a CS-exposed larynx. Specifically, we used a 4-week subacute whole-body CS inhalation mouse model to assess these responses in the laryngeal mucosa upon exposure to low (LD; 1 h/day) and high dose (HD; 4 h/day) CS. Laryngeal tissues were harvested and evaluated using a 254-plex NanoString inflammation panel and neutrophil/macrophage/T-cell immunohistochemistry (IHC). NanoString global and differential gene expression analysis revealed a unique expression profile only in the HD group, with 26 significant differentially expressed genes (DEGs). StringDB KEGG pathway enrichment analysis revealed the involvement of these DEGs with pro-inflammatory pathways including TNF/TNFα and IL-17. Furthermore, inflammatory responses remained inhibited in conjunction with predicted activated states of anti-inflammatory regulators like PPARγ and NFE2L2 upon Ingenuity Pathway Analysis (IPA). Subglottic T-cell levels remained significantly inhibited as corroborated by IPA predictions. Overall, our key findings are consistent with HD exposures being anti-inflammatory and immunosuppressive. Furthermore, the identification of important regulatory genes and enriched pathways may help improve clinical interventions for CS-induced laryngeal diseases.

## Introduction

The larynx is a multifunctional organ involved in voice production, coughing, breathing, and swallowing^1–3^. Located between the upper and lower airways, it is a major target for inhaled toxicants like cigarette smoke (CS). Cigarette smoking is a significant risk factor for multiple laryngeal diseases including Reinke's edema, laryngitis, laryngeal leukoplakia, and laryngeal cancer^4–7^. Notably, laryngeal inflammation is a hallmark clinical feature associated with CS-induced laryngeal diseases^6^. Specifically, adult smokers exhibit edematous vocal folds along with increased vocal fold (VF) mass, irritation, and dehydration^6,8,9^. This affects the vibration of the VF and the quality of voice produced^6,8,9^. CS-induced inflammation can also lead to increased mucus production and impaired mucociliary clearance which causes persistent coughing and throat clearing^10,11^. Although treatment options are available to address some of the effects of cigarette smoking on laryngeal inflammation, irreversible tissue damage can still occur. A current lack of knowledge of the inflammatory processes driving CS-induced disease development impedes proper assessment and treatment.

The laryngeal mucosal surfaces (i.e., epithelium and lamina propria) are the first exposed to harmful external stimuli such as CS. The larynx is lined by ciliated pseudostratified columnar respiratory epithelium except for the VF and epiglottic regions which are lined by stratified squamous epithelium^12^. We have previously characterized tissue remodeling in the laryngeal mucosa after CS exposure (CSE)^12–14^. Morphological alterations to the laryngeal epithelium, as driven by changes to cell proliferation, cell death, epithelial barrier integrity, subglandular hypertrophy, and mucin dynamics were observed^12–14^, consistent with previous reports^15–17^. The mode of action by which CS induces these changes is complex and not well elucidated. As seen in CS-induced pathologies in other parts of the airways and the cardiovascular system^18–21^, the underlying mechanisms likely include alterations to inflammatory immune responses.

Research pertaining to inflammatory immune responses upon CS exposures in other parts of the respiratory tract, such as the lungs, is abundant. Specifically, CSE has been shown to modulate the level of inflammatory cytokines and chemokines as well as the innate and adaptive immune responses, via the signaling cascades regulated by Nuclear factor kappa-B (NF-κB), mitogen-activated protein kinases (MAPK), and signal transducer and activator of transcription (STAT) activators^19,20,22^. Active smoking results in the massive recruitment of innate immune cells, namely neutrophils and macrophages, and it is also known to increase levels of adaptive T-cell populations like CD3+, CD4+, and T-helper 17 (Th17)^19,20,22^. However, data specific to immune response in the laryngeal tissue upon CSE is limited. CSE studies in the larynx have shown elevated levels of individual inflammatory cytokines, like Interleukin-4 (IL-4) in mice^23^ and Tumor necrosis factor-alpha (TNF/TNFα), Interleukin-6 (IL-6) in rats^10^. Contrastingly, TNF/TNFα and IL-6 mRNA and protein levels remained unchanged post CSE in pig larynges, in addition to unaltered levels of other molecules like Interleukin-1 beta (IL-1β), Interleukin-1 receptor 1 (IL-1R1) and Interleukin-12A (IL-12A)^24^. To date, the effect of CSE on inflammatory immune cells in the larynx has rarely been studied. Moreover, there is a paucity of information related to specific pathways in which these inflammatory cytokines and immune cells interact and initiate signaling cascades. The incorporation of pathway-based analysis in CSE studies can shed light on the underlying laryngeal disease mechanisms and facilitate the identification of potential therapeutic targets.

In this regard, the current study seeks to investigate the relationship between cigarette smoking and laryngeal inflammatory immune responses in our previously established CS-injury mouse model^13^. We hypothesized that CS would induce inflammatory responses in the laryngeal mucosa and may give us information about the key inflammatory changes that may play a role in the progression of CS-induced laryngeal diseases. To assess this hypothesis, we exposed mice to two different CSE regimens, namely low dose (LD; 1 h/day) and high dose (HD; 4 h/day) over a period of 4 weeks. A NanoString inflammation panel was utilized to analyze gene expression and signaling pathway enrichments of 248 inflammation-specific genes. Furthermore, we performed an immunohistochemical assessment of inflammatory immune cell infiltration (neutrophil/macrophage/T-cell) into the larynx upon CSE.

## Results

### General effects of cigarette smoking on mice health

Mice in both CSE groups survived 4 weeks of exposure with no obvious health issues. Mice body weight across experimental groups was not significantly different at baseline. Significant body weight reductions were observed in the HD group mice in comparison to control group mice at the end of CS exposures (Supplementary Fig. S1). Also, the average body weight of LD group mice at the exposure end remained similar to their group body weight measured at the experimental start. However, the LD group's average body weight remained significantly lower than the average body weight gained by the control group after 4 weeks.

### NanoString global and differential gene expression analysis

**Figure 1:**
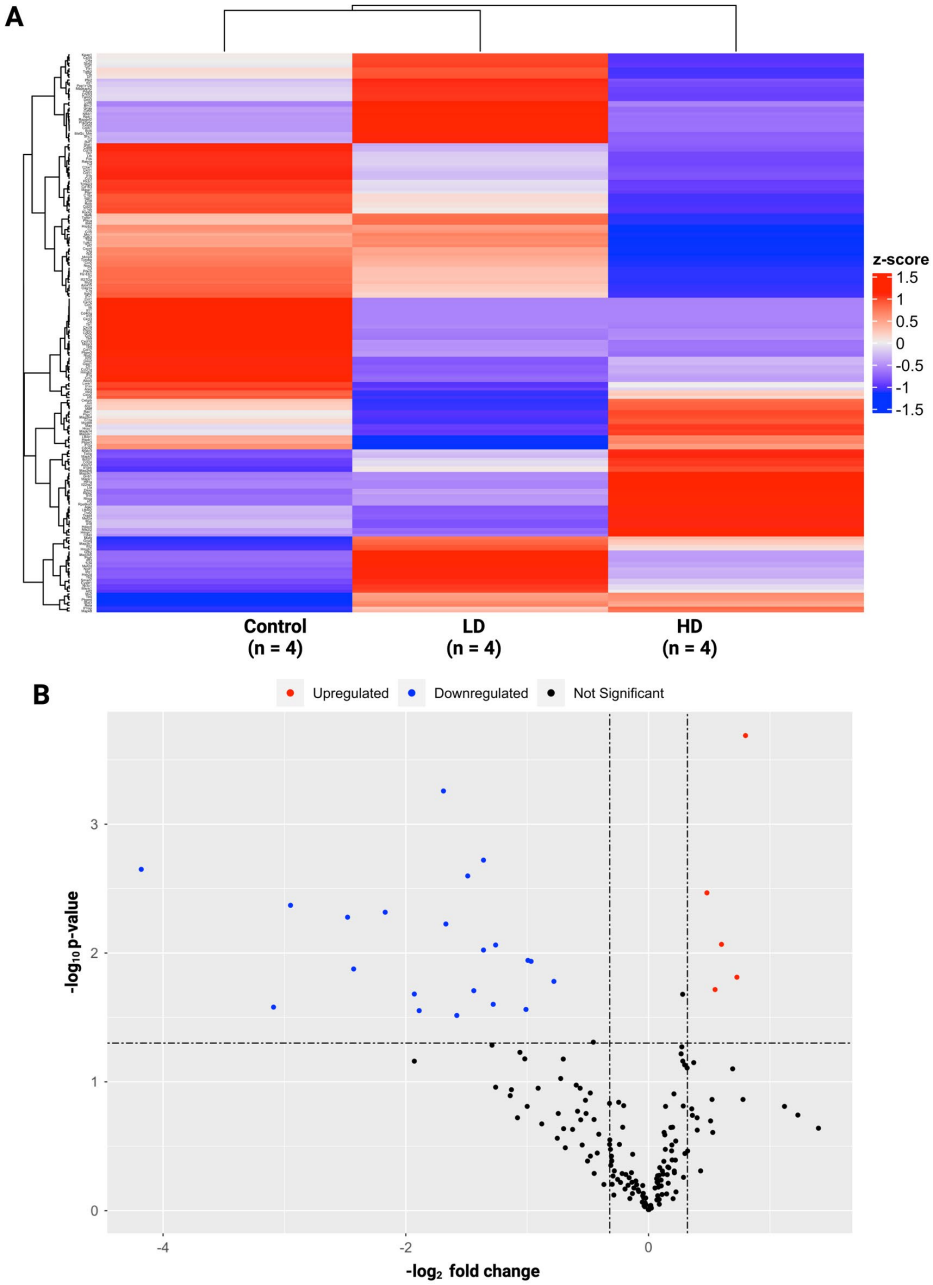
NanoString global and differential gene expression analysis. Upon hierarchical clustering analysis of the NanoString datasets, inflammatory genes in control and LD groups had a higher degree of similarity, whereas these genes were substantially different in the HD group (**A**). Differential expression analysis revealed 26 differentially expressed genes (DEGs) only across HD and control groups (**B**). Specifically, 5 upregulated and 21 downregulated genes were identified as differentially expressed upon HD exposures.

Hierarchical clustering of the NanoString datasets indicated that inflammatory genes in the control and LD groups shared a higher degree of similarity, whereas these genes were considerably different in the HD group (Fig. 1A). No significant differentially expressed genes (DEGs) were identified between LD and control groups based on the statistical significance cutoff levels of p-value ≤ 0.05, FDR < 0.2, and fold change > + 1.25 or fold change < − 1.25 (Supplementary Table S1). A total of 26 genes (5 upregulated and 21 downregulated) were identified as the most significant DEGs between HD and the control groups (Fig. 1B) (Supplementary Table S2).

### StringDB Protein–protein interaction (PPI) network analysis, hub gene identification, and pathway enrichment

**Figure 2:**
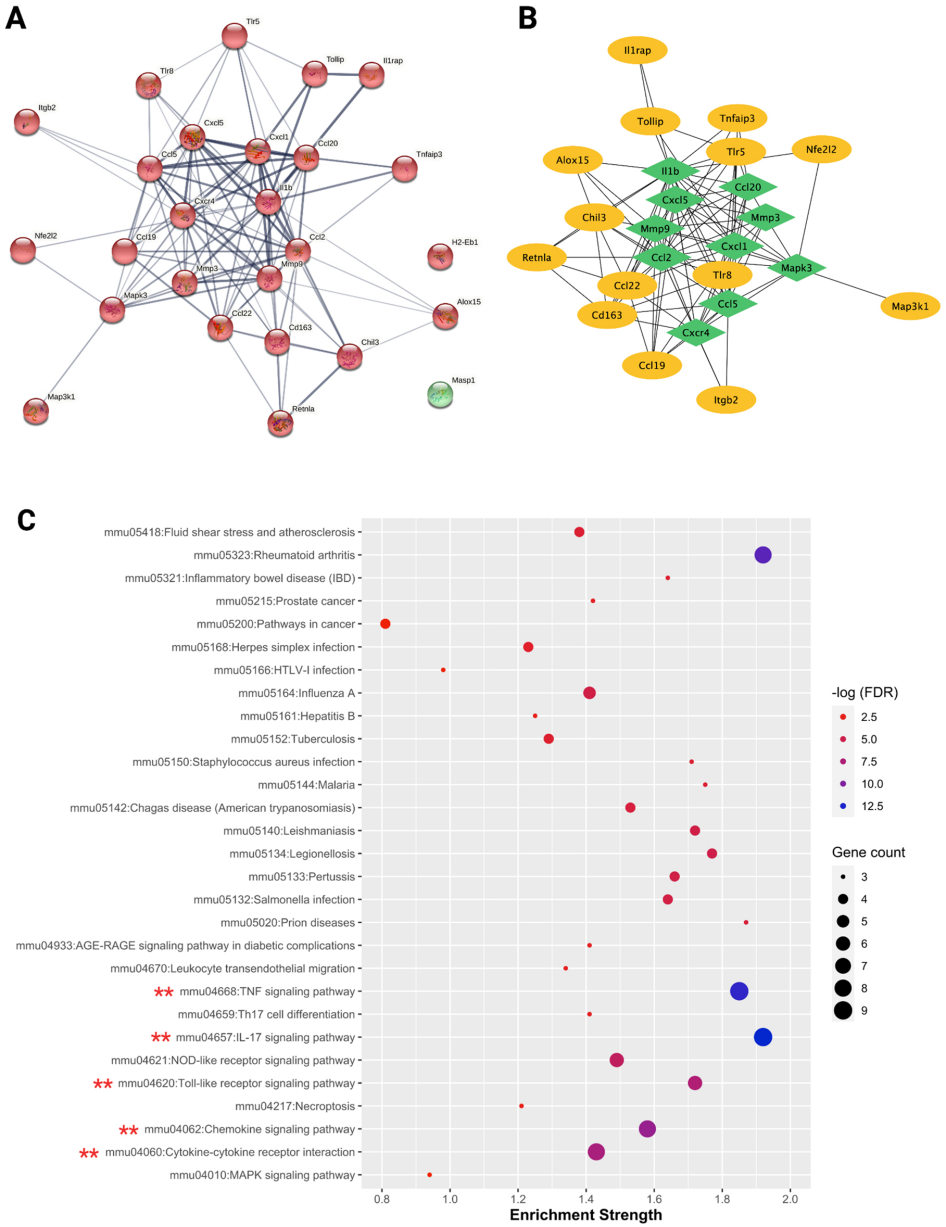
StringDB PPI network analysis and KEGG pathway enrichment. Medium confidence clustered (red) protein–protein interaction network (PPI) was built for HD group DEGs using StringDB (**A**). The Maximal Clique Centrality (MCC) algorithm present in the cytoHubba plugin of the Cytoscape software was used for PPI hub gene identification. MMP9, Cxcl1, Cxcl5, Cxcr4, IL-1β, MMP3, Ccl2, Ccl20, Ccl5, and Mapk3 were the top 10 hub genes identified as indicated by the green diamond-shaped boxes (**B**). KEGG pathway-based enrichment analysis^25–28^ of the StringDB PPI revealed 29 significantly enriched pathways, with the top 5 inflammation-associated pathways as indicated by double, red-colored asterisks (**C**).

A medium confidence protein–protein interaction (PPI) network was built via The Search Tool for the Retrieval of Interacting Genes Database (StringDB) for 26 DEGs identified between HD and control groups, to look at their interactions at a protein level. Within this PPI, one large gene cluster (highlighted in red) was identified (Fig. 2A). The top 10 hub genes identified in this PPI using the Maximal Clique Centrality (MCC) algorithm were Matrix metalloproteinase 9 (MMP9), C-X-C Motif Chemokine Ligand 1 (Cxcl1), C-X-C Motif Chemokine Ligand 5(Cxcl5), C-X-C Motif Chemokine Receptor 4 (Cxcr4), IL-1β, Matrix metalloproteinase 3 (MMP3), C-C Motif Chemokine Ligand 2 (Ccl2), C-C Motif Chemokine Ligand 20 (Ccl20), C-C Motif Chemokine Ligand 5 (Ccl5), and Mitogen-Activated Protein Kinase 3 (Mapk3) (Fig. 2B). Out of the 10 hub genes, 7 important inflammatory cytokine-related genes, namely, a pro-inflammatory cytokine, IL-1β, and chemotactic cytokines (chemokines), Cxcl1, Cxcl5, Cxcr4, Ccl2, Ccl20, and Ccl5 were identified. In addition, hub genes from the family of matrix metalloproteinases, MMP9 and MMP3 were also identified within the top 10 hub genes. Kyoto Encyclopedia of Genes and Genomes (KEGG)^25–28^ pathway enrichment analysis of HD group StringDB PPI interactions identified 29 significantly enriched pathways. Among these pathways, the top 5 inflammation-related pathways were the Interleukin-17 (IL-17) signaling pathway, TNF/TNFα signaling pathway, Chemokine signaling pathway, Cytokine-cytokine receptor interaction, and Toll-like receptor (TLR) signaling pathway (Fig. 2C) (Supplementary Table S3).

### StringDB PPI hub gene expression validation

**Figure 3:**
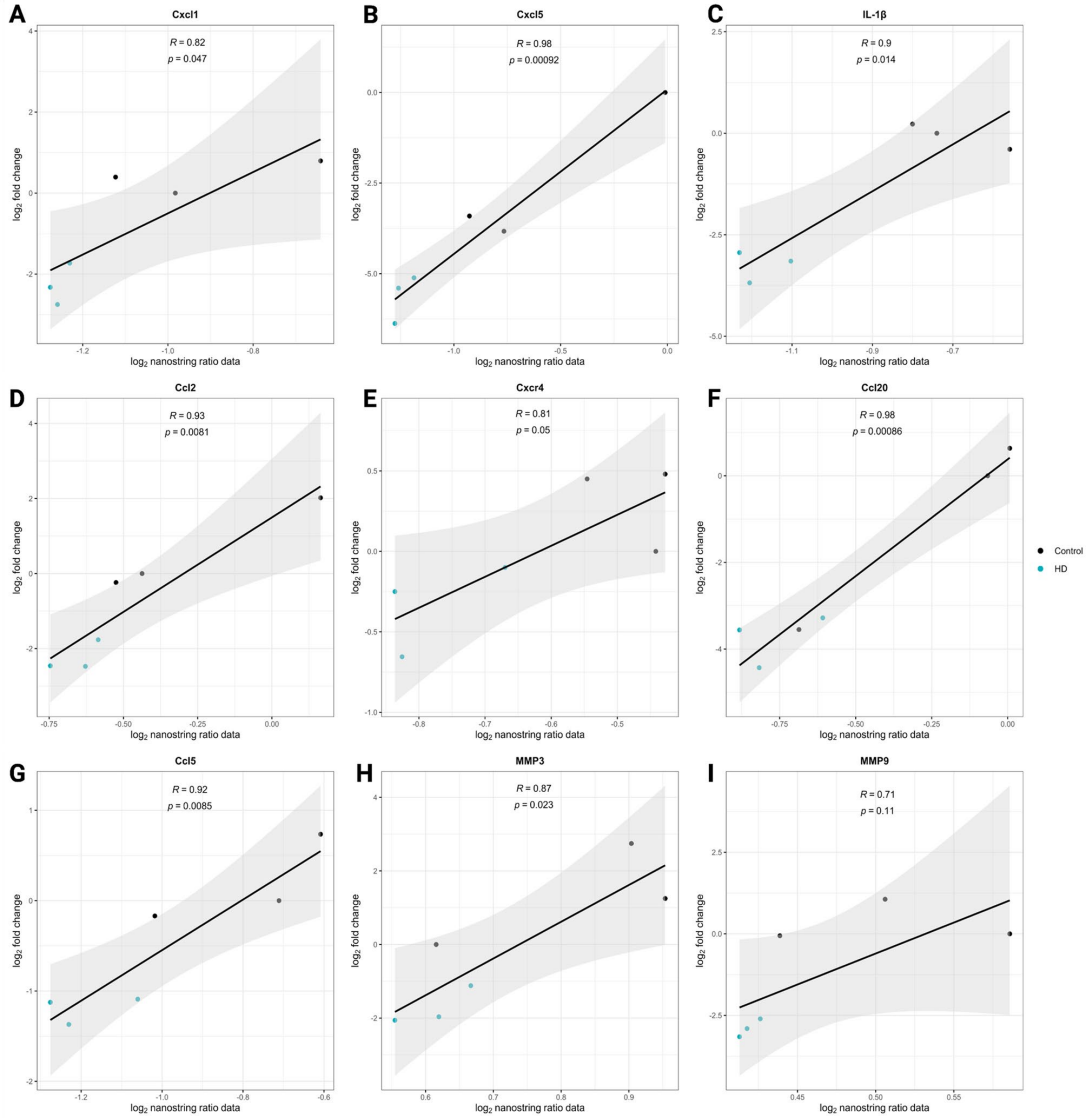
Inter-platform correlation between NanoString and qPCR assays. Log_2_ fold change from real-time PCR (qPCR) analysis was subjected to pairwise Pearson correlation against log_2_ fold changes obtained from NanoString analysis for key downregulated hub genes including, Cxcl1 (**A**), Cxcl5 (**B**), IL-1β (**C**), Ccl2 (**D**), Cxcr4 (**E**), Ccl20 (**F**), Ccl5 (**G**), MMP3 (**H**), and MMP9 (**I**). High to very high positive correlation between their NanoString and qPCR mRNA expression levels was observed for all these hub genes. Pearson correlation coefficient ‘R’ values ranged from 0.71 to 0.98 for all genes.

The 7 key inflammatory cytokine-related hub genes previously identified were significantly enriched in the aforementioned top 5 inflammation-related KEGG pathways^25–28^, with all 7 genes enriched in the cytokine-cytokine receptor interaction pathway (Supplementary Table S3). Matrix metalloproteinases, MMP9 and MMP3 were specifically enriched within the top 2 significantly enriched pathways related to IL-17 and TNF/TNFα signaling (Supplementary Table S3). Quantitative real-time polymerase chain reaction (qPCR) analysis of these hub genes revealed a significant decrease in gene expression for Cxcl1, Cxcr4, IL-1β, Ccl5, and MMP9 (Supplementary Fig. S2), thereby complementing the significantly downregulated pattern of these genes as derived by NanoString analysis (Supplementary Fig. S3; Supplementary Table S2). Despite the significant downregulation of Cxcl5, Ccl2, Ccl20, and MMP3 genes (Supplementary Fig. S3; Supplementary Table S2), their fold changes remained non-significant upon qPCR analysis, showing a tendency towards decreased gene expression (Supplementary Fig. S2). Furthermore, inter-platform correlation analysis between NanoString and qPCR data provided an outcome of high to very high positive correlation for all these genes (Fig. 3A–I).

### Identification of top upstream regulators and biofunctions via Qiagen’s Ingenuity Pathway Analysis (IPA)

**Figure 4:**
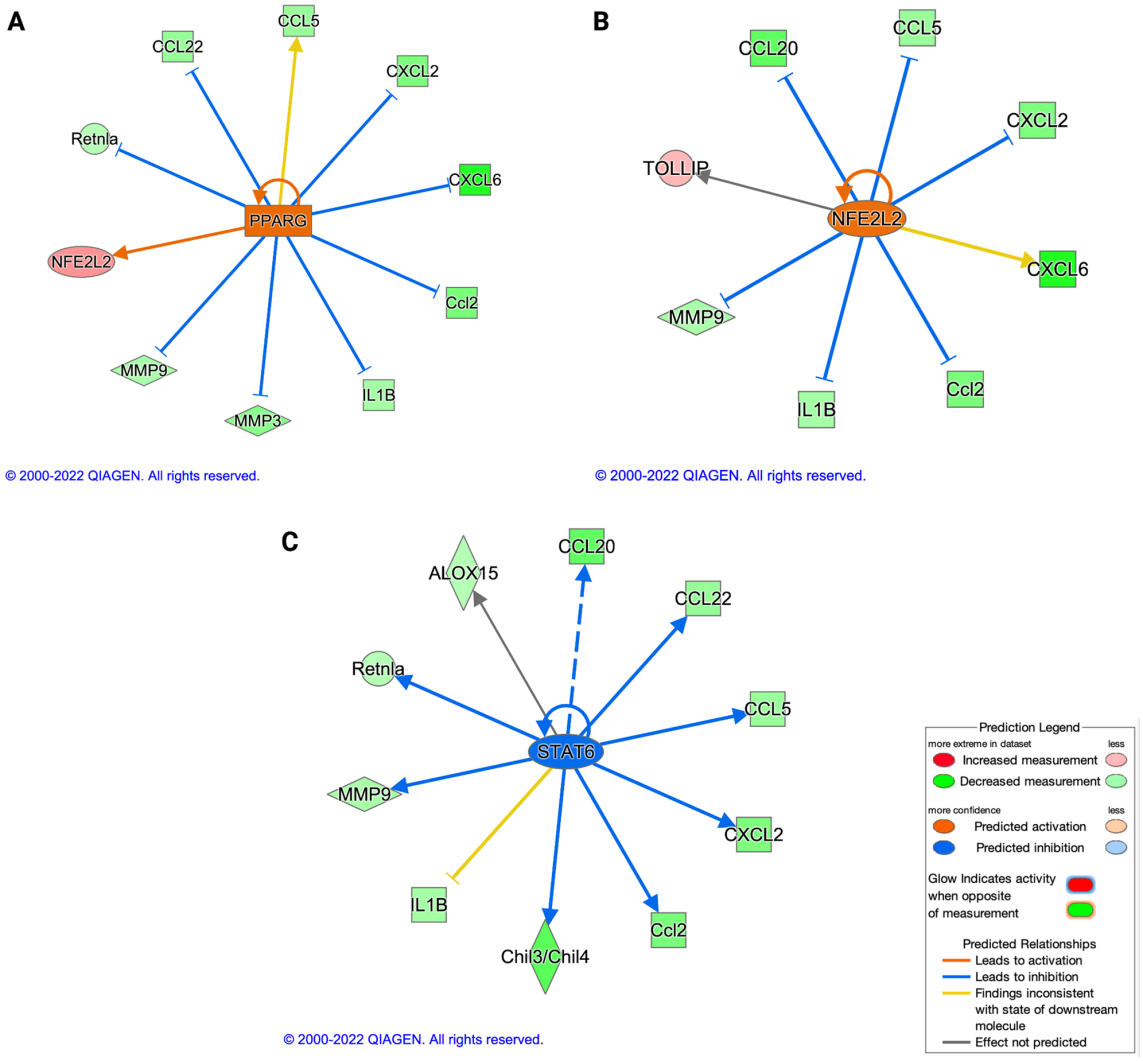
Significant upstream regulators identified by Qiagen’s Ingenuity Pathway Analysis (IPA). Peroxisome proliferator-activated receptor gamma (PPARγ) (**A**) and Nuclear factor, Erythroid 2 like 2 (NFE2L2) (**B**) were predicted to be significantly activated regulators whereas Signal transducer and activator of transcription 6 (STAT6) (**C**) was predicted to be significantly inhibited by IPA. Genes from the NanoString dataset in the HD group which are regulated by these individual upstream regulators are depicted in each of these panels. The level of significance was set as p-value of overlap < 0.05, with z-score < − 2 or z-score > 2.

The most significant upstream regulators identified by Ingenuity Pathway Analysis (IPA) for the HD vs control group DEGs are Peroxisome proliferator-activated receptor gamma (PPARγ) (Fig. 4A), Nuclear factor, Erythroid 2 like 2 (NFE2L2/Nrf2) (Fig. 4B), and Signal transducer and activator of transcription 6 (STAT6) (Fig. 4C). Specifically, PPARγ and NFE2L2 were predicted to be activated regulators (z-score > 2; p-value of overlap < 0.05), whereas STAT6 was predicted to be inhibited (z-score < − 2; p-value of overlap < 0.05) (Supplementary Table S4).

**Figure 5:**
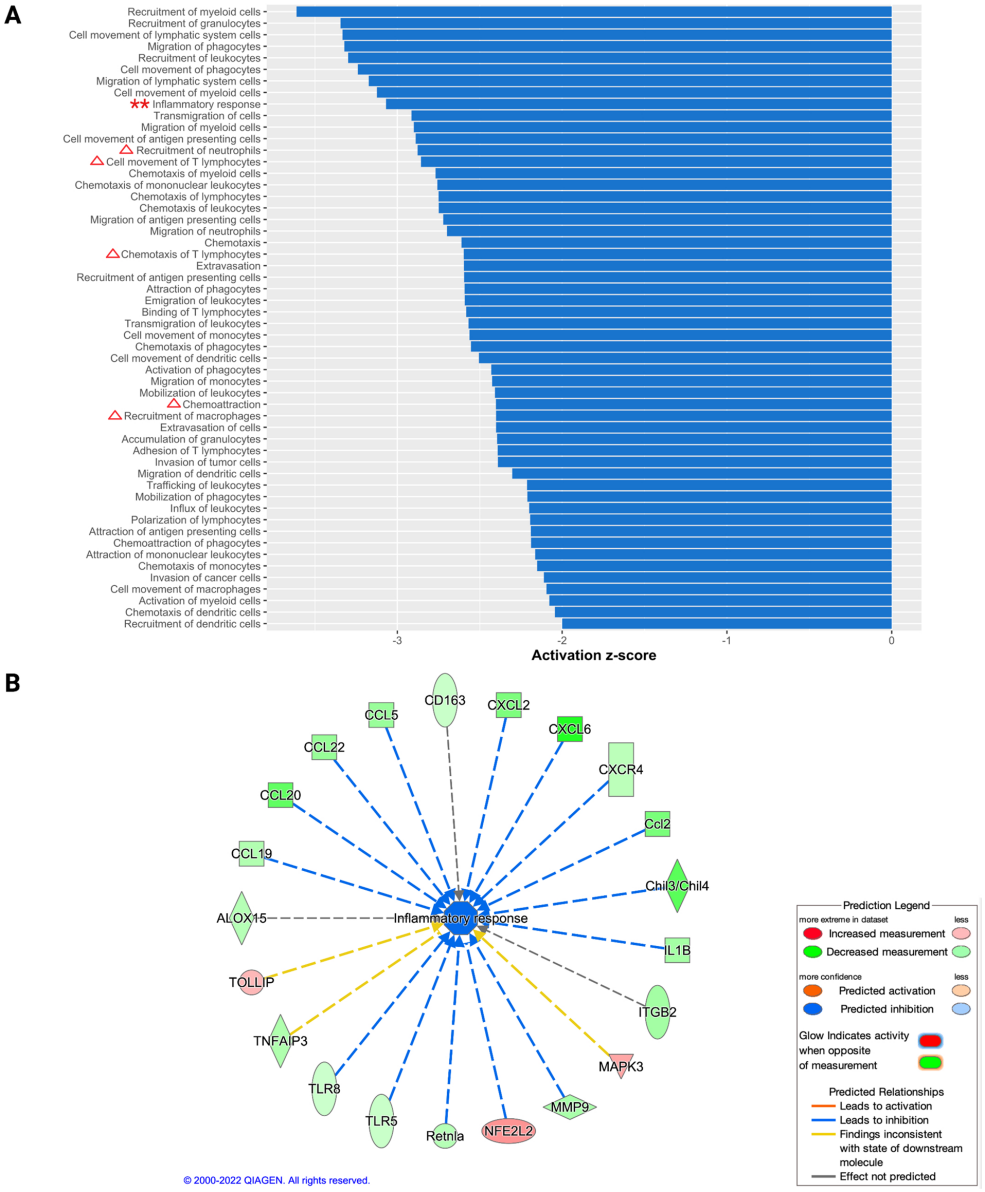
Significant biofunctions identified by Qiagen’s Ingenuity Pathway Analysis (IPA). Out of the total 55 bio functions identified to be significantly inhibited by IPA (**A**), inflammatory response was within the top 10 biofunctions identified as indicated by double, red-colored asterisks. Immune cell recruitment and chemotactic functions related to neutrophils, macrophages, and T-cells were predicted to be decreased as well as indicated by red-colored triangles (**A**). Genes from the NanoString dataset in the HD group which contribute towards decreased inflammatory responses are depicted in panel (**B**). The level of significance was set as p-value of overlap < 0.05, with z-score < − 2 or z-score > 2.

In terms of biological functions and diseases, there was no activated function/disease predicted by IPA for these DEGs. A total of 55 biofunctions were predicted to be significantly inhibited (z-score < − 2; p-value of overlap < 0.05) by IPA (Fig. 5A), which were predominantly classified into the following categories: cell-to-cell signaling and interaction, cellular movement, inflammatory response, hematological system development, and function and immune cell trafficking (Supplementary Table S5). Inflammatory responses were within the top 10 biofunctions inhibited (Fig. 5A, B). Furthermore, the recruitment and chemotactic functions of immune cells like neutrophils, macrophages, and T-cells were predicted to be decreased as well (Fig. 5A).

### Validation of IPA upstream regulator and biofunctional or disease prediction outcomes

**Figure 6:**
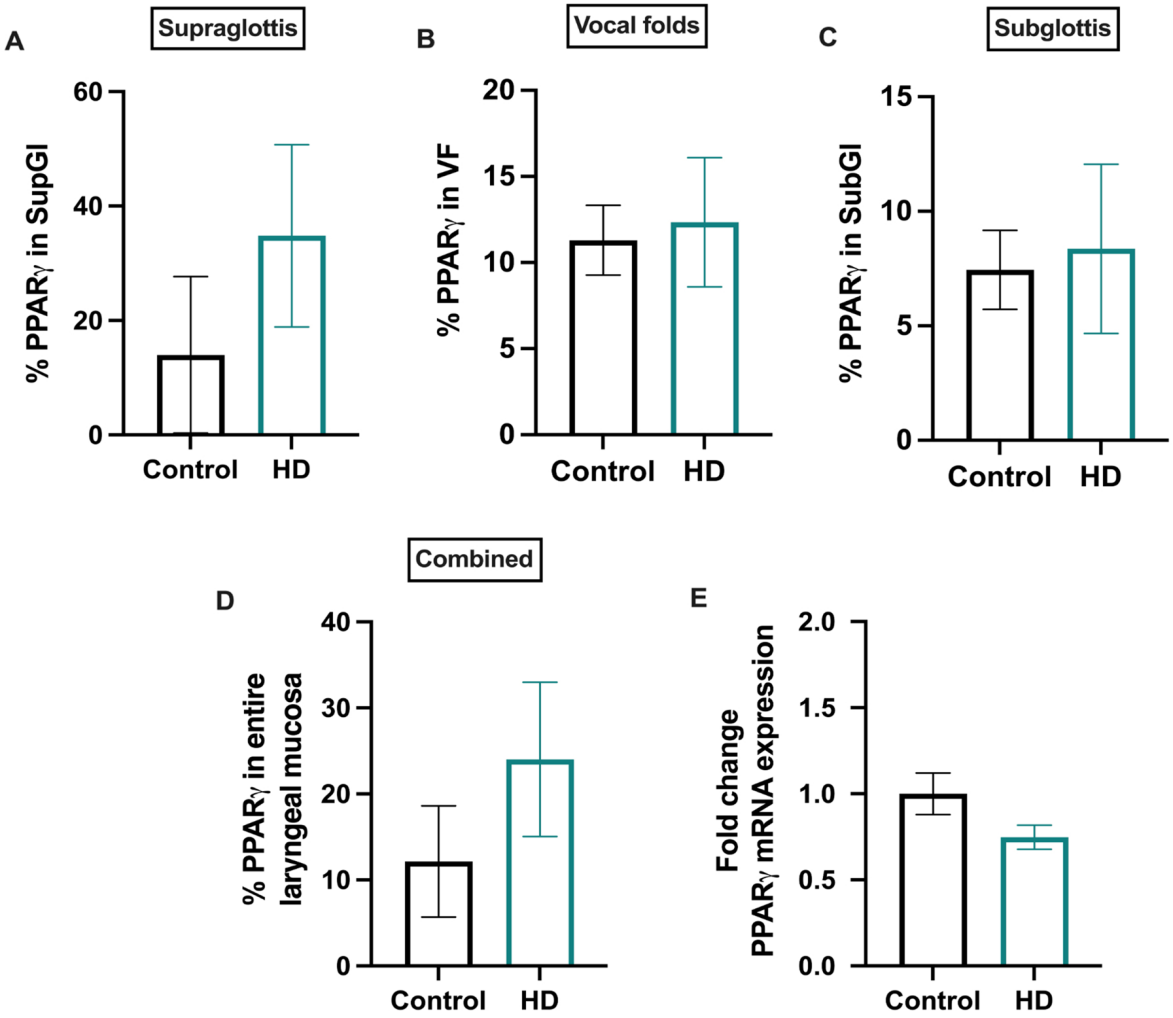
Quantification of PPARγ in the laryngeal mucosa. Immunohistochemical evaluations revealed that PPARγ expression levels remained non-significant in the HD group in comparison to the control group in supraglottis (**A**), vocal folds (**B**), subglottis (**C**), and when averaged across entire laryngeal mucosa including the aforementioned regions (**D**). mRNA expression levels of PPARγ were also unaltered between HD and control groups (**E**). Gene expression was normalized to the reference gene, Gusb. Bar plots are represented as mean with SEM.

IHC and qPCR techniques were used to validate IPA upstream regulators and biofunctional or disease prediction outcomes. An automated digital immunohistochemical analysis was performed via QuPath in the supraglottic, VF, and subglottic laryngeal regions to quantify expression levels of PPARγ, the top activated regulator predicted by IPA. Despite exhibiting a strong inclination towards increased expression in the laryngeal mucosal region, notably, the supraglottic region, IHC analysis indicated that PPARγ levels remained non-significant between control and HD groups (Fig. 6A–D; Supplementary Fig. S4). Moreover, mRNA expression levels of PPARγ were also unaltered (Fig. 6E). Additionally, qPCR analysis of the second predicted activated regulator, NFE2L2 was also performed. Like PPARγ, mRNA expression levels of NFE2L2 were not significantly changed, despite showing a trend of mild increase in gene expression upon HD CS exposures (Supplementary Fig. S5).

Apart from NFE2L2 and the hub genes including IL-1β, Cxcl5 or Cxcl6^29^, Ccl2, Ccl5, MMP3, and MMP9 as prioritized by IPA to be regulated by PPARγ as seen in Fig. 4A, other genes such as chemokines like C-C Motif Chemokine Ligand 22 (Ccl22) and C-X-C Motif Chemokine Ligand 2 (Cxcl2) and chemotactic immune cell modulator like Resistin-like molecule alpha (Retnla) were also identified. mRNA expression levels of these three genes were also estimated. Our outcomes revealed a significant decrease in Ccl22 gene expression in the HD group, whereas Cxcl2 and Retnla levels were not significant, despite having a strong predisposition towards decreased gene expression upon HD exposures (Supplementary Fig. S5).

**Figure 7:**
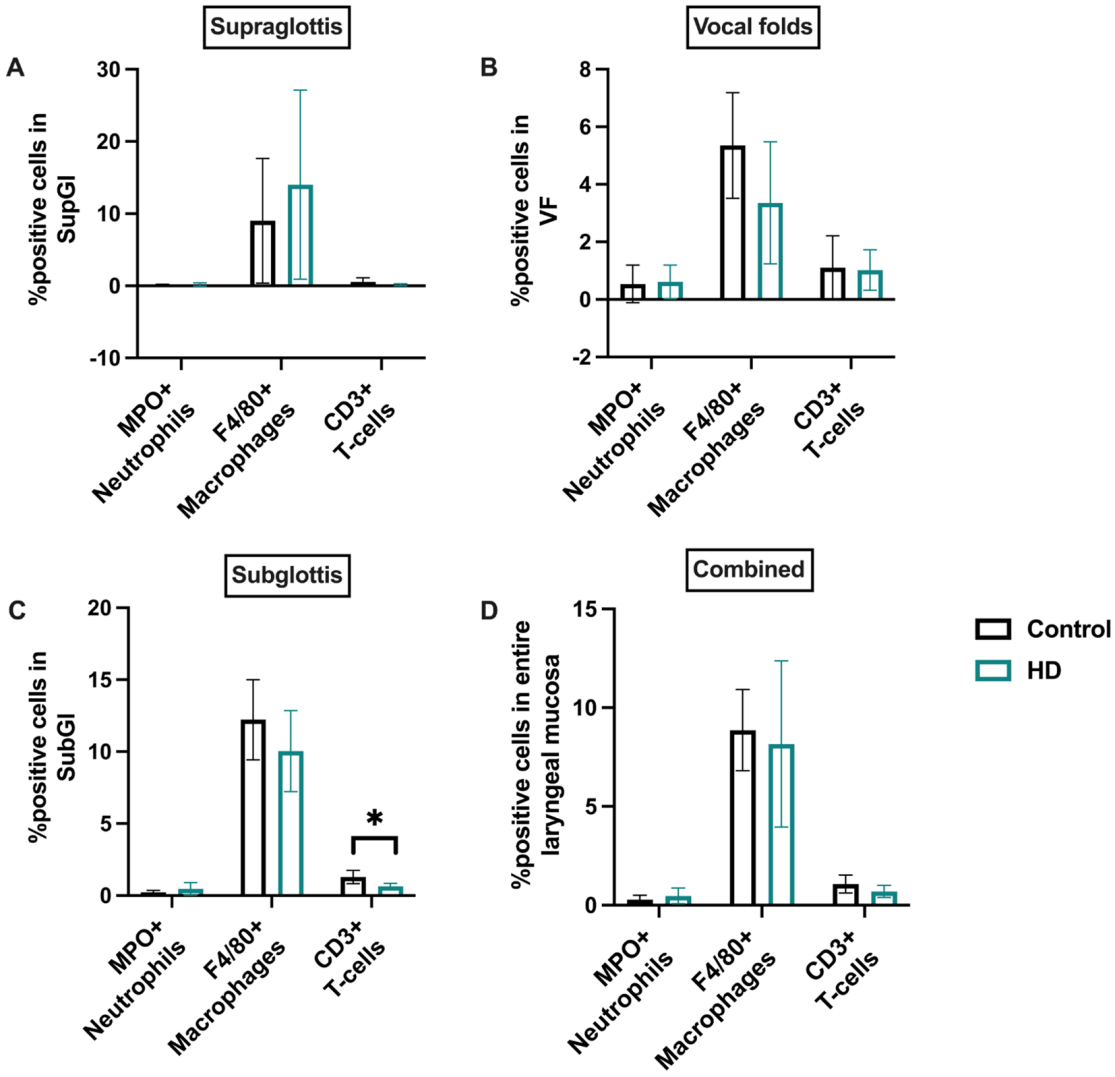
Quantification of immune cells in the laryngeal mucosa. HD CSE had no impact on the MPO + neutrophils and F4/80+ macrophages levels in the supraglottis (**A**), vocal folds (**B**), subglottis (**C**), and when averaged across entire laryngeal mucosa including the aforementioned regions (**D**). CD3 + T-cells were significantly decreased in the subglottic laryngeal regions (**C**). Despite the observed significant downregulation, CD3+ T-cells were unaltered in the supraglottic (**A**) and vocal fold (**B**) regions and when averaged across entire laryngeal mucosa (**D**). Bar graphs show the mean with SEM. * p ≤ 0.05.

Based on the IPA predictions of decreased immune cell recruitment/chemotactic responses, an automated digital immunohistochemical analysis of immune cell infiltrates were also performed in supraglottic, VF, and subglottic laryngeal regions. The outcomes indicated no major differences in the levels of MPO+ neutrophils and F4/80+ macrophages in all three laryngeal regions (Fig. 7A–D) (Supplementary Fig. S6; Supplementary Fig. S7; Supplementary Fig. S8). Although average CD3+ T-cell levels taken across these three regions remained unaltered between the control and HD groups, individual regional examinations show that HD CS exposures significantly decrease T-cell levels only in the subglottic laryngeal regions and not in the VF or supraglottic regions (Fig. 7A–D) (Supplementary Fig. S6; Supplementary Fig. S7; Supplementary Fig. S8). In general, neutrophil and T-cells levels were minimally detected in all three laryngeal regions.

Immune cells were also quantified via qPCR using specific marker genes including S100A8 and S100A9 for neutrophils and macrophages and CD3-epsilon polypeptide (CD3ε) for all T-cell populations. Gene expression levels for these three genes had no major changes, although a very strong proclivity towards decreased mRNA expression in the HD group is to be noted (Supplementary Fig. S9).

## Discussion

The larynx has a significant role as a gatekeeper of airway immunology^3,30^. Given its strategic location at the upper-lower airway divergence, the larynx is highly predisposed to environmental toxicants like CS, and its subsequent immunological responses can determine disease onset and progression. Despite the high incidences of CS-related laryngeal diseases, the underlying inflammatory mechanisms remain largely unexplored. In this current study, mice were exposed to LD (1 h/day) and HD (4 h/day) CS for 4 weeks in order to evaluate inflammatory and immune cell infiltrate responses. The results demonstrated that only HD, but not LD CS was anti-inflammatory after 4 weeks of subacute exposure. Our findings also indicate that it significantly decreased T-cell levels only in subglottic regions, whereas the levels of early inflammatory immune cell participants like neutrophils and macrophages remained unchanged.

Upon analysis via IPA, PPARγ was predicted to be an activated upstream regulator of the HD DEGs, and inflammatory responses were predicted to be inhibited. Moreover, IHC evaluations also showed a strong inclination towards elevated PPARγ levels upon HD CS exposures, although overall levels remained non-significant in comparison to the controls, which is corroborated by PPARγ qPCR outcomes as well. Previous in vitro literature has demonstrated that HD CS exposures are anti-inflammatory and immunosuppressive in human peripheral blood mononuclear cells^31,32^. Notably, higher dose CS exposures can activate Peroxisome proliferator-activated receptor (PPAR) signaling^31^. PPAR ligands, including PPARγ, impart anti-inflammatory properties by transrepression of NF-κB signaling, a classic pro-inflammatory CS-related pathway^33–37^. PPARγ activation reduces CS-induced inflammation via NF-κB regulation in the lungs, shedding light on PPARγ as a promising therapeutic target to reverse lung inflammation^38^. PPARγ can also specifically inhibit the production of inflammatory cytokines including TNF/TNFα, IL-1β, and IL-6^34,39,40^. Correspondingly, we also saw a significant downregulation of IL-1β mRNA levels in this study. Furthermore, PPARγ has been implicated as a vital player in resolving inflammation via skewing the direction of macrophage polarization towards M2 (T helper 2 cells [Th2] related) anti-inflammatory phenotype^34,41^. In this study, we utilized a pan macrophage marker F4/80 for estimating macrophage levels. Our outcomes show no major changes to macrophage levels in the larynx, contrary to their expected increased levels upon CSE as reported earlier in the lower airways^20,42,43^. In this study, it is possible that a resolution of inflammation or a shift from pro-inflammatory (T helper 1 cells [Th1]/M1) to anti-inflammatory (Th2/M2) phenotype might have occurred in response to PPARγ activation upon HD CS exposures. In order to validate this, we will need continued efforts to gain a deeper understanding of the effects of PPARγ signaling on laryngeal inflammation upon acute and chronic CS exposures. The incorporation of a PPARγ transgenic mouse model and detailed assessments of M1 and M2 macrophages in future CS inhalation studies can expand this effort.

Most of the top 10 hub genes identified from the HD group StringDB PPI in this study were downregulated inflammatory cytokines and chemokines, namely IL-1β, Cxcl1, Cxcl5, Cxcr4, Ccl2, Ccl20, and Ccl5. In addition to enrichment in cytokine-cytokine receptor interaction and chemokine signaling pathways, StringDB KEGG pathway-based analysis^25–28^ also revealed significant enrichment of these DEGs in inflammatory signaling pathways including IL-17 and TNF/TNFα signaling. Furthermore, hub genes MMP3 and MMP9 were also enriched in IL-17 and TNF/TNFα signaling pathways. Overproduction of IL-17 and TNF/TNFα has been implicated in CS-associated inflammation in various studies^20,44–49^. In conjunction with IL-1β signaling^50^, both IL-17 and TNF signaling can promote inflammation mainly via neutrophil and macrophage accumulation in the lungs^48,51^. In this study, we saw chemokine and chemokine receptor downregulation specific to neutrophils: Cxcl1, Cxcl5, Cxcr4, Ccl5^52,53^; macrophages: Ccl2^54,55^, in addition to T-lymphocyte associated chemokines, Ccl20^52,56^. Moreover, we also observed downregulated patterns in MMP3 and MMP9 gene expression in this study, which are mainly produced by neutrophils and macrophages at inflammatory conditions^57–59^. In terms of direct quantification of immune cells, our qPCR and IHC evaluations predominantly indicated unaltered levels of these immune cells post HD exposures, except for significantly decreased CD3+ T-cell levels observed only in the subglottic laryngeal regions upon IHC. Interestingly, previous reports indicate that an agonist of the upstream regulator molecule, PPARγ identified in this study can reduce IL-17 signaling via inhibition of NF-κB signaling activity in a mouse model of asthma^60^. It is possible that the downstream inflammatory effects of IL-17 signaling were attenuated alongside TNF/TNFα, IL-1β, and NF-κB signaling upon PPARγ activation in our study, thereby limiting its effects on immune cell recruitment as well. Incidentally, we identified that majority of the downregulated hub genes like IL-1β, Cxcl5 or Cxcl6^29^, Ccl2, Ccl5, MMP3, and MMP9 were prioritized by IPA to be PPARγ-regulated. Previous literature provides evidence that activation of PPARγ not only inhibits inflammatory cytokine production^34,39,40^, but also inhibits MMPs like MMP9 in the airways^61^. Furthermore, other PPARγ-regulated chemokines specific to neutrophils and macrophages like Cxcl2 and Ccl22^52,62^ and chemotactic immune cell modulator like Retnla^63–66^ from IPA also show indications of downregulation upon HD exposures. Therefore, future studies will be needed to further examine the effects of this regulatory molecule on these specific pathways/molecules in a larynx upon CSE.

Another pathway significantly enriched in this study was TLR signaling. We observed significant downregulation of TLR5 and TLR8 (Supplementary Table S2). All other TLRs in the NanoString panel remained unchanged (Supplementary Table S2). TLRs are important pathogen recognition receptors that play a major role in eliciting inflammatory innate immune responses against invading pathogens and toxicants like CS^20,67^. The effects of CS on TLR responses are equivocal^20^. While it has been well established that CS can promote inflammation in the lungs via TLR2/4 activated signaling^68–71^, there are other studies that show that CS can dampen responses of other TLRs like TLR3/5 in the lungs, thereby enhancing susceptibility to microbial/viral infections^67,72–74^. Whether the reduction in TLR5 response post-exposure in this study can lead to an increased risk of infection in the larynx awaits investigation. Furthermore, prior literature also provides compelling evidence of an existing negative feedback loop between TLR signaling and PPARγ which can modulate inflammation^75–77^. To advance our understanding, comprehensive evaluations of TLR-mediated laryngeal inflammatory immune responses are imperative.

Our findings via IPA also predicted another upstream regulator, NFE2L2 or Nrf2 to be activated in response to HD CS exposures. Moreover, qPCR and NanoString fold changes demonstrated a tendency towards upregulation of the NFE2L2 gene (Supplementary Table S2; Supplementary Fig. S5). Corroborating with our data, NFE2L2 activation was also observed in short-term CS-exposed human alveolar macrophages^78^. NFE2L2 is a key transcription factor, which stimulates the expression of the gene, antioxidant response element (ARE) under severe oxidative stress conditions like CSE^79,80^. NFE2L2-ARE signaling is pivotal in maintaining intracellular redox homeostasis and preventing inflammation or excessive tissue damage^78^, especially after repetitive CSE. Previous in vivo reports show that NFE2L2 deletion leads to severe emphysema and oxidative stress, upon prolonged CSE^81,82^. Strikingly similar to PPARγ, the NFE2L2-ARE signaling cascade also limits the redox-sensitive pro-inflammatory NF-κB signaling pathway and production of inflammatory cytokines^83^. In fact, NFE2L2 has also been shown to induce PPARγ expression under hypoxic conditions in the lungs, indicating that PPARγ is a downstream effector molecule of the NFE2L2-ARE signaling response^36,83^. Furthermore, synergistic effects of these molecules have been implicated in the activation of antioxidant genes^84^, suggesting that these molecules have antioxidant properties in addition to being anti-inflammatory. It is well established that in the lungs, CS-induced oxidative stress and intracellular redox imbalances can activate cell signaling cascades regulated by NF-κB, MAPK, and Activator protein-1 (AP-1)^85^. These signaling pathways are involved in the activation of multiple inflammatory mediators^20,86^, which eventually promote inflammatory immune cell recruitment. In this study, we only observed significantly decreased subglottic CD3+ T-cell levels upon IHC and saw no major changes in neutrophils or macrophages. However, downregulation of inflammatory cytokine/chemokines related to these immune cells like Cxcl2 and key hub genes including IL-1β, Cxcl5 or Cxcl6^29^, Ccl2 and Ccl5 were observed. Coincidentally, all these molecules were predicted to be commonly regulated by both NFE2L2 and PPARγ upon IPA regulatory analysis. Hence, it is probable that there was a mutual activation of NFE2L2 and its effector molecule, PPARγ upon HD CSE, and their interactions antagonized CS-induced oxidative stress, the resulting inflammatory immune cell responses, and various signaling cascades. Upcoming studies will need to explore the role of the positive feedback mechanisms between PPARγ and NFE2L2 on the laryngeal inflammatory response upon CSE.

It is currently unknown which specific toxicants present in CS induced the potential anti-inflammatory and immunosuppressive effects observed upon subacute HD CSE. The effects of CS on host immune responses are complex and bimodal in nature. Individual CS toxicants can cause both pro-inflammatory and immunosuppressive effects^20,22,87^. In this study, it is unknown whether anti-inflammatory and immunosuppressive effects were direct damages caused only by the immunosuppressive CS toxicants or if their cumulative effects outweighed the effects of pro-inflammatory components together at 4-week HD exposure levels. Also, it is noteworthy that it has been earlier reported that CS components in the vapor phase are not immunosuppressive when compared to particulate phase CS components^87^. Future studies are needed to evaluate the effects of these specific CS toxicants in both particulate and vapor phases on the laryngeal inflammatory and other immunological responses upon short- and long-term CSE.

We recognize that this study was designed to evaluate CS-induced responses from a specific timepoint of 4 weeks, which is considered subacute based on the Organization for Economic Co-operation and Development (OECD) inhalation toxicity guidelines^88^. While earlier in vivo studies have demonstrated inflammation in the airways upon response to CSE in a similar timeframe^89,90^, our findings in this study indicates that 4 weeks of CSE does not induce a severe inflammatory disease-like condition in the mouse larynx. Specifically, our IHC data showed significant decreased levels of certain inflammation-specific immune cells like T-cells, whereas non-significant decreasing tendencies were observed upon qPCR along with similar expression patterns from neutrophil- and macrophage-specific immune cell markers. It is currently unknown whether these outcomes are a compensatory response to initiate inflammation resolution or true immunosuppression. If it is an inflammation resolution response, then it is possible that we missed detecting these cells at 4 weeks as they are known to arrive at the inflammatory sites within sec-hours-days of contact with an external stimulus^91,92^. Contrarily, continual immunosuppression can eventually lead to carcinogenesis^93,94^. Although this study did not focus on mechanisms related to carcinogenesis, our previous work in this CS-injury mouse model^13^ has shown increases in the levels of known laryngeal cancer prognostic makers, p63 and Ki67^95^ upon HD exposures. To further investigate whether these responses are compensatory or true immunosuppression with carcinogenic potential, timeline is an important factor to consider. In order to characterize a timeline of events leading up to sustained inflammation and carcinogenesis as seen with CS-induced human laryngeal diseases, comprehensive short- and longer-term assessments are required in future studies.

There are some limitations of this study to be addressed. The overall objective of this initial study was to obtain a broad perspective on the laryngeal inflammatory responses to CSE and to generate hypotheses for upcoming studies. Consequently, we did not focus on specific enriched pathways. We now have potential molecular/pathway targets for functional validation in future studies. In addition, auxiliary evaluations of anti-inflammatory mediators, and oxidative/antioxidant gene markers could have strengthened our bioinformatic anti-inflammatory and antioxidant outcomes. Subsequent studies should be directed at examining these profiles in a CS-exposed larynx. In terms of IPA upstream regulator (PPARγ and NFE2L2) and biofunctional (immune cell) data validation, we do not see a strong correlation between bioinformatic, IHC, and qPCR outcomes. Possible explanations for these technical variations can be arising from complex steps involved in bioinformatics and algorithm-based digital pathology analysis, data normalization, amplification, protein vs mRNA transcript level abundance, subjective interpretation regarding the regions of interest selected for IHC, and small sample sizes. Secondly, these responses maybe time dependent themselves. For example, mRNA quantification of certain important molecules predicted to be regulated by PPARγ and NFE2L2 by IPA, show expected patterns of desired up- or downregulation, necessary for activating their respective upstream regulators. However, this study couldn’t capture significant activation of both PPARγ and NFE2L2 at 4 weeks with routinely used techniques such as qPCR and or IHC. It is to be noted here that IPA’s predictions with our current NanoString gene dataset were based on the pre-existing trends from literature extensively mined from various data sources ranging across different experimental timelines or designs. It is possible that if these expected gene expression patterns persist with time, we may be able to capture stronger activation responses of these regulators. Given that this reiterates the importance of time dependency, it is necessary to investigate the activation/inactivation states of these specific molecules or responses in future studies with additional timepoints. Also, we assessed a small subset of representative inflammatory immune cells including neutrophils, macrophages, and T-cells. CS affects a wide range of innate and adaptive immune cells that contribute to the inflammatory process such as natural killer cells, dendritic cells, mast cells, B-cells, CD4 + helper T-cells, CD8 + cytotoxic T-cells, and regulatory T- cells^20,87^. The impact of CS on these immune cells in the larynx awaits investigation. Furthermore, in vivo inhalation toxicology studies often measure the levels of nicotine biomarkers in biological samples like urine or serum. We did not perform these measurements in our study. Prior studies also indicate that mice lose body weight in response to successful in vivo CS inhalation^12,96–98^. Correspondingly, we observed in this study that the HD mice exhibited a significant body weight reduction in comparison to the controls. In our future research, we aim to integrate these nicotine biomarker analyses in order to establish in vivo smoking status.

To our knowledge, this is the first study to evaluate the impact of LD and HD CS exposures on inflammation in the mouse larynx. Specifically, this subacute, 4-week study indicates that only higher doses of CS induce responses consistent with anti-inflammatory and immunosuppressive conditions in the larynx. This study also provides insights into the possible underlying inflammatory pathway mechanisms of disease development and sets the stage for future investigation of therapeutic interventions. Furthermore, the inclusion of bulk or single-cell RNA sequencing in combination with spatial transcriptomics in forthcoming research can help us gain a thorough understanding of laryngeal inflammation and other immunological responses. Also, given the gaining popularity of the use of electronic cigarettes and heat-not-burn products, our upcoming studies may benefit from including these devices as a comparative group, to further our understanding of their effects on the health of the larynx.

## Methods

### Animal and housing care

Male C57Bl/6 mice,15 weeks in age were purchased from Charles River laboratories. Mice were housed separately in ventilated cages with normal chow and water ad libitum, in the Stanford Veterinary Service Center. The room was maintained at 23 °C, 60% relative humidity, and with a 12 h light/12 h dark cycle. Mice were continuously monitored for any possible health issues and body weights were measured thrice weekly. All experimental procedures with mice were performed in accordance with the relevant guidelines and regulations as reviewed and approved by the Stanford University Institutional Animal Care and Use Committee (approval number: APLAC-31912). The study was designed and performed in compliance with the ARRIVE guidelines 2.0.

### Experimental design

**Figure 8:**
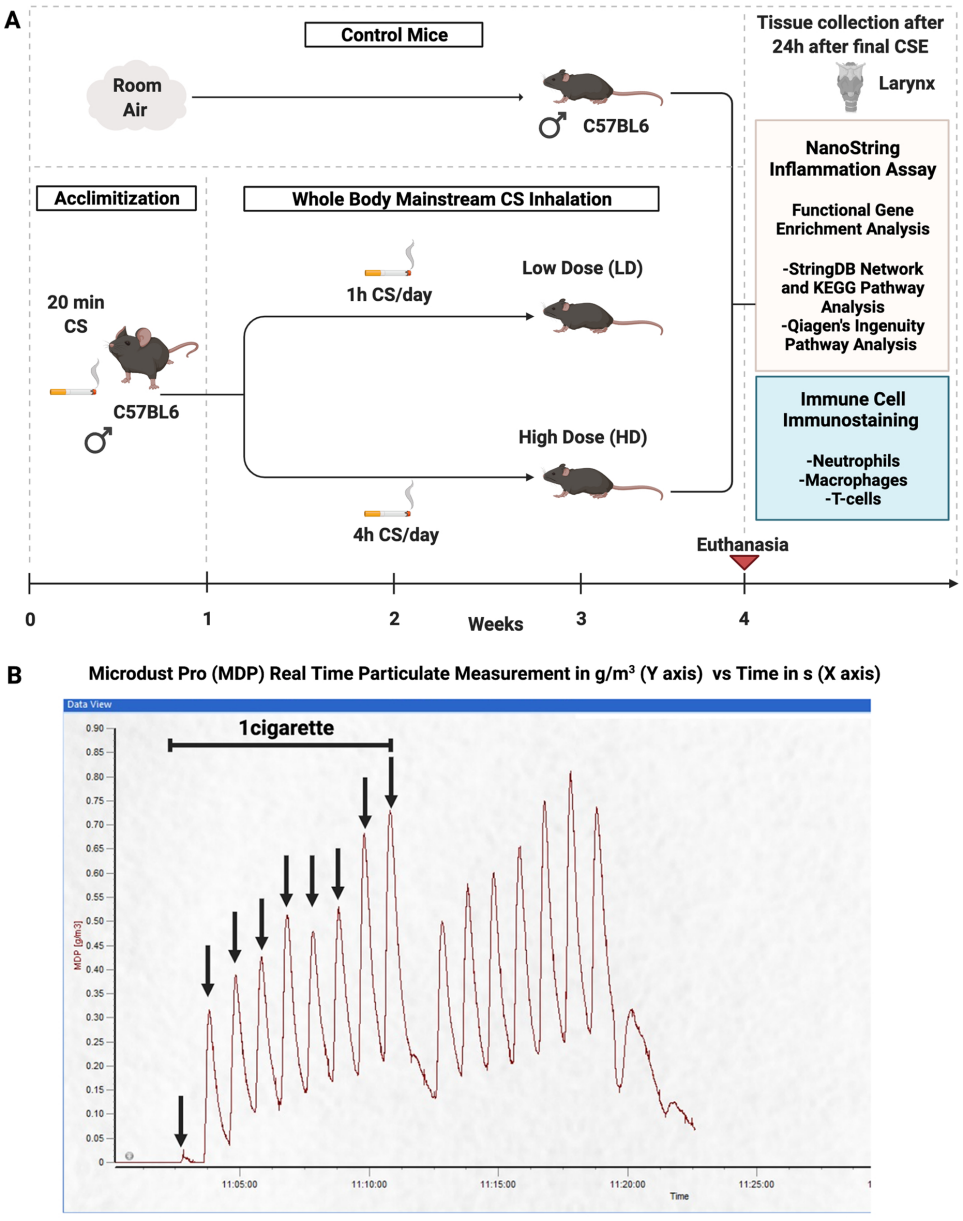
Experimental design. Adult male C57Bl/6 were assigned to control, low dose (LD), and high dose (HD) groups (**A**). Control mice were air exposed. LD and HD mice were exposed to 1 h and 4 h of cigarette smoke (CS) per day respectively, for a total of 4 weeks, 5 days/week. LD and HD mice were acclimated to 20 min of CS per day for a week prior to the start of exposures. Laryngeal tissues were harvested 24 h after the final CS exposure (CSE) at week 4 for NanoString analysis and associated network and pathway-based functional gene enrichment analysis. Harvested larynges were also processed for immunohistochemical staining. CS exposures were monitored in real-time by using a probe, Microdust Pro (**B**). Each recorded peak represents a puff and a total of 9 puffs were recorded per 3R4F cigarette.

Mice were randomly allocated to control (n = 10), LD (n = 12), and HD CSE groups (n = 12) (Fig. 8A). All CS exposures were via whole-body administration. LD mice were exposed to CS for 1 h/day. HD mice were exposed to 4 h of CS per day in 1–1.5 h increments, with room air breaks. Earlier studies in the airways and specifically in the larynx, have shown that 1–4 h/day of CSE is sufficient to induce histopathological changes^12,13,99,100^. LD and HD CS exposures were performed for 5 days/week, M-F for a total of 4 weeks. Based on inhalation toxicity guidelines regulated by OECD, 4 weeks is considered subacute exposure^88^. Previous work in rats has shown inflammatory phenotypic outcomes in the airways upon CS exposures for 4 weeks^89^ or 5 weeks^90^. Before 4 weeks of CSE, the mice in both groups were acclimatized to ~ 20 min of CS per day, for 5 days (M-F). The exposure chamber concentration (610 µg/L) and delivered dose (mg/body weight) per cigarette (5.4882 mg/kg) were similar to our previous study^13^. Control mice were left exposed to HEPA-filtered room air conditions.

### Cigarette smoke generation

Mainstream CS from Kentucky 3R4F reference cigarettes was generated using inExpose smoking equipment (SCIREQ, Montreal, QB, Canada), consistent with our previous studies^12–14^. Exposure conditions were per the Federal Trade Commission (FTC)/International Standard Organization (ISO) standards (ISO 1991). Essential parameters were a puff duration of 2 s, a puff volume of 35 mL, a puff count of 9 per cigarette, and a puff frequency of 1 puff/min. Based on these puff characteristics, LD and HD group mice were exposed to 9 and 27 cigarettes/day respectively for 4 weeks. CS exposures were monitored in real-time using a probe, Microdust pro (Casella, UK) (Fig. 8B).

### Animal euthanasia, tissue collection, and processing

Mice were euthanized by cervical dislocation under deep anesthesia by isoflurane 24 h after the final CSE. Larynges were harvested and split between histological and RNA-related analyses. For histological analyses, larynges were fixed in 4% paraformaldehyde overnight at 4 °C and transferred to 70% ethanol (n = 5 control, n = 6 each in both CSE groups). HistoWiz, Inc. (http://www.histowiz.com; Brooklyn, NY, USA) processed tissues for paraffin embedding and performed coronal section microtomy per established methods^12–14,101,102^. 5 µm coronal sections were used for immunohistochemical assessment of immune cell infiltrates. The remaining larynges across experimental groups (n = 5 control, n = 6 each in both CSE groups) were assigned for RNA extraction and stored in RNAlater solution (Catalog no. AM7020, Invitrogen, CA, USA) at − 80 °C until RNA isolation.

### Total RNA isolation and reverse transcription

Whole larynges were thawed and hemisected. Mucosal and submucosal tissues, starting from the glottis until the lower end of subglottic regions were harvested from the hemisected larynges. Total RNA was extracted according to the RNeasy Mini Kit (Qiagen, Germantown, MD, USA). RNA quantity and quality (A_260_/A_280_) were estimated using the Nanodrop 2000 spectrophotometer (Thermo Fisher Scientific, Rockford, IL, USA). Samples from control (n = 1), low and high dose groups (n = 2 each) with poor RNA yield and A_260_/A_280_ values were eliminated from subsequent analysis. RNA integrity number (RIN) values were computed for remnant RNA elutes (n = 4 per group). Isolated RNA from all samples had an acceptable RIN value > 8 (Supplementary Fig. S10). Subsequently, reverse transcription was performed with 0.6 μg of RNA per reaction according to the manufacturer’s instructions in the iScript™ complementary DNA (cDNA) synthesis kit (Catalog no. 170–8890; Bio-Rad, Hercules, CA, USA).

### Inflammatory gene expression assay and analysis with NanoString technology

Laryngeal mRNA was analyzed with a NanoString nCounter mouse inflammation v2 panel (248 inflammation-specific genes + 6 internal reference controls; Catalog no. XT-CSO-MIN2-12, NanoString Technologies, Seattle, WA, USA). Isolated RNA (~ 250 ng) from all biological samples across control, LD, and HD groups (n = 4 each) were specific to this panel. The number of occurrences of these unique bar-coded probes was counted by NanoString’s nCounter instrument for every sample. Raw data counts were obtained for further analysis.

NanoString’s data analysis application software, nSolver version 4.0 was utilized for the analysis of raw data counts. All experimental samples were annotated based on their exposure conditions. The background threshold was calculated as 12 using the recommended formula: Mean negative probe counts + 2 [standard deviation of negative probe counts]. Probes that had expression < 12 were eliminated from the analysis. The dataset was normalized using positive control and housekeeping probes. The resulting normalized and annotated grouped data were used for subsequent analysis. Global gene expression across different experimental conditions was assessed via complete linkage hierarchical clustering with Euclidean distance metrics using annotated grouped data in R. Differential gene expression was performed on the normalized dataset via advanced analysis module version 2.0 on nSolver. Benjamini–Hochberg false discovery rate (FDR) correction was employed to compute adjusted p-values. Comparisons between two independent groups (a) HD vs control and (b) LD vs control were conducted. Genes that passed a modest statistical cutoff^103^ of p-value ≤ 0.05, FDR < 0.2, and fold change > + 1.25 or fold change < − 1.25 were identified to be significantly upregulated or downregulated.

### PPI network analysis using StringDB and Cytoscape

The interaction of DEGs at the protein level was analyzed by using StringDB (string-db.org). Medium confidence PPI clustered networks with a minimum score of 0.4 and Markov Cluster Algorithm inflation parameter as 2 were built. KEGG pathway enrichment analysis^25–28^ was performed, with FDR ≤ 0.05 and minimum gene count as 3 considered to be statistically significant^104^. Within the PPIs, the most interactive gene nodes, known as hub genes, were identified using the cytoHubba plugin^105^ in the open-source software application, Cytoscape (http://www.cytoscape.org/; version 3.8.2). Out of the 12 different topological algorithms present in the cytoHubba plugin in the Cytoscape platform^105^, the MCC algorithm has a better execution in hub gene identification^106^ and we used this topological algorithm to identify the top 10 hub genes.

### Upstream regulators and biofunctions identification via IPA

Upstream analysis and diseases and biofunctions modules were run on the DEGs via core analysis in IPA. All genes in the normalized NanoString dataset were used as the background reference set to compute enrichment. Upstream regulators and biological functions with Fisher’s exact p-value of overlap < 0.05 and z-score > 2 or z-score < − 2 were identified to be significant.

### qPCR validation of StringDB PPI hub gene and IPA prediction outcomes

qPCR was performed with CFX96 Real-Time PCR Machine (Bio-Rad) to validate NanoString gene expression levels of the key hub genes identified from StringDB PPI analysis. Gene expression for important molecules from IPA upstream regulator and biofunctional or disease prediction outcomes were also validated via qPCR. Mouse-specific primer sequences were designed based on NanoString target probe sequences using ApE, a plasmid editor (Table 1)^107^. qPCR was carried out using iTaq™ Universal SYBR® Green Supermix (Catalog no.1725120; Bio-Rad, Hercules, CA, USA) in addition to template cDNA and specific primers. All qPCR experiments were conducted in duplicates with a sample size of n = 3 per group. Relative quantitative analysis of gene expression was completed using the standard comparative Ct method (2^−ΔΔCt^) by using the recorded raw Ct values of the gene of interest and housekeeping gene^108^. Using RefFinder, Gusb was selected as the most stable reference gene for qPCR normalization based on expression stability assessment among the other common candidate reference genes, Gapdh, Hprt, and Tubb5, present in the NanoString panel^109,110^.

**Table 1:** Primers sequences for qPCR.

Target gene symbol	Primer sequences (5′-3′)		References
Cxcl1	FP	TGCTAGTAGAAGGGTGTTGTGCG	–
	RP	ACACAGCCTCCCACACATGTCC	
Cxcl5	FP	GGTCCACAGTGCCCTACG	^113^
	RP	GCGAGTGCATTCCGCTTA	
Ccl2	FP	TCTTCAGCACCTTTGAATGTGAAGT	–
	RP	TGGTCACTCCTACAGAAGTGCT	
Cxcr4	FP	GTTTCAATTCCAGCATATAATGGTGGG	–
	RP	GCCCTTGGAGTGTGACAGC	
Ccl20	FP	GCAGCAAGCAACTACGACTGTT	–
	RP	CACAAGCTTCATCGGCCATCTG	
Ccl5	FP	CATATGGCTCGGACACCA	–
	RP	ACACACTTGGCGGTTCCT	
IL-1β	FP	GTTGATTCAAGGGGACATTAGGCAGC	–
	RP	AATGAAAGACCTCAGTGCGGGC	
MMP3	FP	TCTTTGTGAAAGGAAGTGCTTTGTTCA	–
	RP	TGAACATCCTTTGACAACTTGACGTTG	
MMP9	FP	CCTCTACAGAGTCTTTGAGTCCGG	–
	RP	CCGTCCTTGAAGAAATGCAGAGC	
PPARγ	FP	TTTCAAGGGTGCCAGTTTCG	^114^
	RP	ATCCTTGGCCCTCTGAGATGAG	
NFE2L2	FP	CTGAACTCCTGGACGGGACTA	^115^
	RP	CGGTGGGTCTCCGTAAATGG	
Ccl22	FP	CCAAGAATCAACTTCCACCCCTCTT	–
	RP	CTTCGACTATCAGCTACACAGGCAAG	
Cxcl2	FP	GGTGGGGGTGGGGACAAATAG	–
	RP	GCTGTTCTACTCTCCTCGGTGC	
Retnla	FP	CCTGCTGGGATGACTGCTA	–
	RP	CAAGTATCTCCACTCTGGATC	
S100A8	FP	CCCGTCTTCAAGACATCGTTTG	^116^
	RP	ATATCCAGGGACCCAGCCCTAG	
S100A9	FP	CCCTGACACCCTGAGCAAGAAG	^116^
	RP	TTTCCCAGAACAAAGGCCATTGAG	
CD3ε	FP	CCTCCTAGCTGTTGGCACTT	–
	RP	ACTGTCTAGAGGGCACGTCA	
Gusb	FP	AATACGTGGTCGGAGAGCTCATC	–
	RP	TGGCGAGTGAAGATCCCCTT	

### Immunohistochemistry

Standard IHC procedures to stain PPARγ, Myeloperoxidase/MPO+ neutrophils, F4/80+ macrophages, and CD3+ T-cells were performed by HistoWiz, Inc (histowiz.com). For PPARγ staining, the sample size was n = 3 for both groups. For all immune cell stains, sample sizes were n = 5 for the control group and n = 6 for the HD group. Stained slides were scanned by Histowiz using an Aperio AT2 microscope (Leica Biosystems, Germany). These scanned virtual slides were used to perform an automated digital analysis of 3,3'-Diaminobenzidine (DAB) positive immune cell counts via QuPath version 0.3.0. For this analysis, the mid-membranous regions in the right and left vocal folds were first traced at 20x (Supplementary Fig. S11). The entire supraglottic region located immediately above the VF region was also traced at 20x (Supplementary Fig. S11). In terms of the right and left subglottic regions, the entire subglottic regions (including epithelial and subglandular regions), starting from the lower end of VF until the first tracheal ring were traced at 10x (Supplementary Fig. S11). Positive immune cell nuclei or cytoplasmic and or membrane staining was estimated in these regions by using the positive cell detection analysis tool. Positive PPARγ staining was detected using average brown DAB staining within the nucleus using Nucleus: DAB OD mean. For cytoplasmic and or membranous immune cell stains, Cell: DAB OD mean was used for detection^111^. The resulting data were expressed as % positive (number of positive detections / total number of cell detections in the traced area in µm^2^) in the entire supraglottic region and as averaged across the right and left sides of both VF and subglottis.

### Statistical analysis

Statistical tests for body weight, IHC, and qPCR analysis were performed using GraphPad Prism version 9.0. Data comparisons were made between control, LD, and HD exposure groups by using (a) two-way analysis of variance (ANOVA) for bodyweight pre/post CSE comparisons and (b) unpaired two-tailed Student t-test for qPCR and IHC analyses. All bar graphs are represented as means ± standard error of mean (SEM). p-value ≤ 0.05 was considered statistically significant. All statistical analyses pertaining to NanoString global and differential gene expression, network analysis, and IPA are described above. Heatmap, volcano, dot and scatter plots were plotted using RStudio version 1.4.1717. Inter-platform correlation between NanoString and qPCR gene expression data of the selected key hub genes was performed via Pearson correlation in RStudio version 1.4.1717. The strength of correlation as indicated by Pearson correlation coefficient R was interpreted according to previously published guidelines^112^.

## Supplementary Information

Supplementary Figures.

Supplementary Tables.

## Data Availability

The datasets generated and/or analyzed during the current study are available in the Gene Expression Omnibus (GEO) repository, [GSE208079].

## Abbreviations

ANOVA: Analysis of variance
ARE: Antioxidant response element
AP-1: Activator protein-1
Ccl2: C-C Motif Chemokine Ligand 2
Ccl5: C-C Motif Chemokine Ligand 5
Ccl20: C-C Motif Chemokine Ligand 20
Ccl22: C-C Motif Chemokine Ligand 22
cDNA: Complementary DNA
CS: Cigarette smoke
CSE: Cigarette smoke exposure
Cxcl1: C-X-C Motif Chemokine Ligand 1
Cxcl2: C-X-C Motif Chemokine Ligand 2
Cxcl5: C-X-C Motif Chemokine Ligand 5
Cxcr4: C-X-C Motif Chemokine Receptor 4
DEGs: Differentially expressed genes
FDR: False discovery rate
HD: High dose
IHC: Immunohistochemistry
IL-1β: Interleukin-1 beta
IL-1R1: Interleukin-1 receptor 1
IL-4: Interleukin 4
IL-6: Interleukin 6
IL-12A: Interleukin-12A
IL-17: Interleukin-17
IPA: Ingenuity pathway analysis
KEGG: Kyoto encyclopedia of genes and genomes
LD: Low dose
MMP3: Matrix metalloproteinase 3
MMP9: Matrix metalloproteinase 9
MAPK: Mitogen-activated protein kinases
Mapk3: Mitogen-Activated Protein Kinase 3
MCC: Maximal clique centrality
MPO: Myeloperoxidase
NFE2L2/Nrf2: Nuclear factor, Erythroid 2 like 2
NF-κB: Nuclear factor kappa-B
OECD: Organization for Economic Co-operation and Development
PPAR: Peroxisome proliferator-activated receptor
PPARγ: Peroxisome proliferator-activated receptor gamma
PPI: Protein–protein interaction
qPCR: Quantitative real-time polymerase chain reaction
Retnla: Resistin-like molecule alpha
STAT: Signal transducer and activator of transcription
STAT6: Signal transducer and activator of transcription 6
StringDB: Search tool for the retrieval of interacting genes database
Th17: T-helper 17
TLR: Toll-like receptor
TNF/TNFα: Tumor necrosis factor alpha
VF: Vocal fold
