# Supplementary material for: Modulation of mouse laryngeal inflammatory and immune cell responses by low and high doses of mainstream cigarette smoke
Source: Sci Rep. 2022 Nov 4;12:18667. doi: 10.1038/s41598-022-23359-7 (PMC9636197; doi:10.1038/s41598-022-23359-7)
Supplement: Supplementary file 1 — Supplementary Figures. [file 41598_2022_23359_MOESM1_ESM.docx]

**Supplementary Figures**

**Modulation of mouse laryngeal inflammatory and immune cell responses by low and high doses of mainstream cigarette smoke**

Meena Easwaran^1,2^, Joshua D. Martinez^1^, Juyong Brian Kim^2^, Elizabeth Erickson-DiRenzo^1^

^1^Department of Otolaryngology-Head and Neck Surgery, Stanford University School of Medicine, Stanford, CA, USA.

^2^Department of Cardiovascular Medicine, Stanford University School of Medicine, Stanford, CA, USA.

***Correspondence:**

Elizabeth Erickson-DiRenzo Ph.D., CCC-SLP

Assistant Professor, Division of Laryngology

Department of Otolaryngology-Head & Neck Surgery

Stanford University School of Medicine

801 Welch Road, Stanford, CA 94305-5739

Office: 650-498-9051

Email: edirenzo@stanford.edu


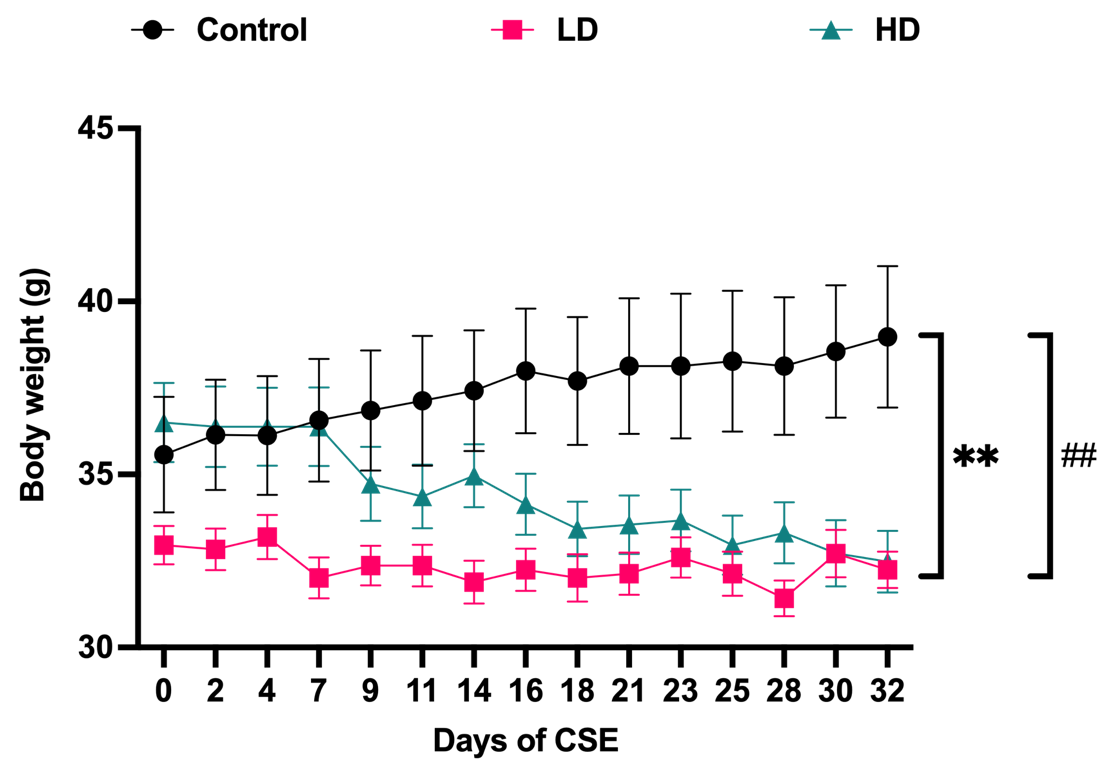


**Supplementary Figure S1. Mice body weight.** Control, LD, and HD experimental group mice did not significantly differ in body weights before the start of CS exposures (Day 0). Body weight of mice in the HD group was significantly lower than the body weight gained by control mice at the end of CS exposures (Day 32). Despite no evident changes to the LD group mice body weight from exposure start to end, their body weight remained significantly lower than the body weight gained by control mice at exposure end. Mean with SEM is depicted in the line graphs. * significance between control and HD. # significance between control and LD. ** p -value ≤ 0.01 and ## p -value ≤ 0.01.


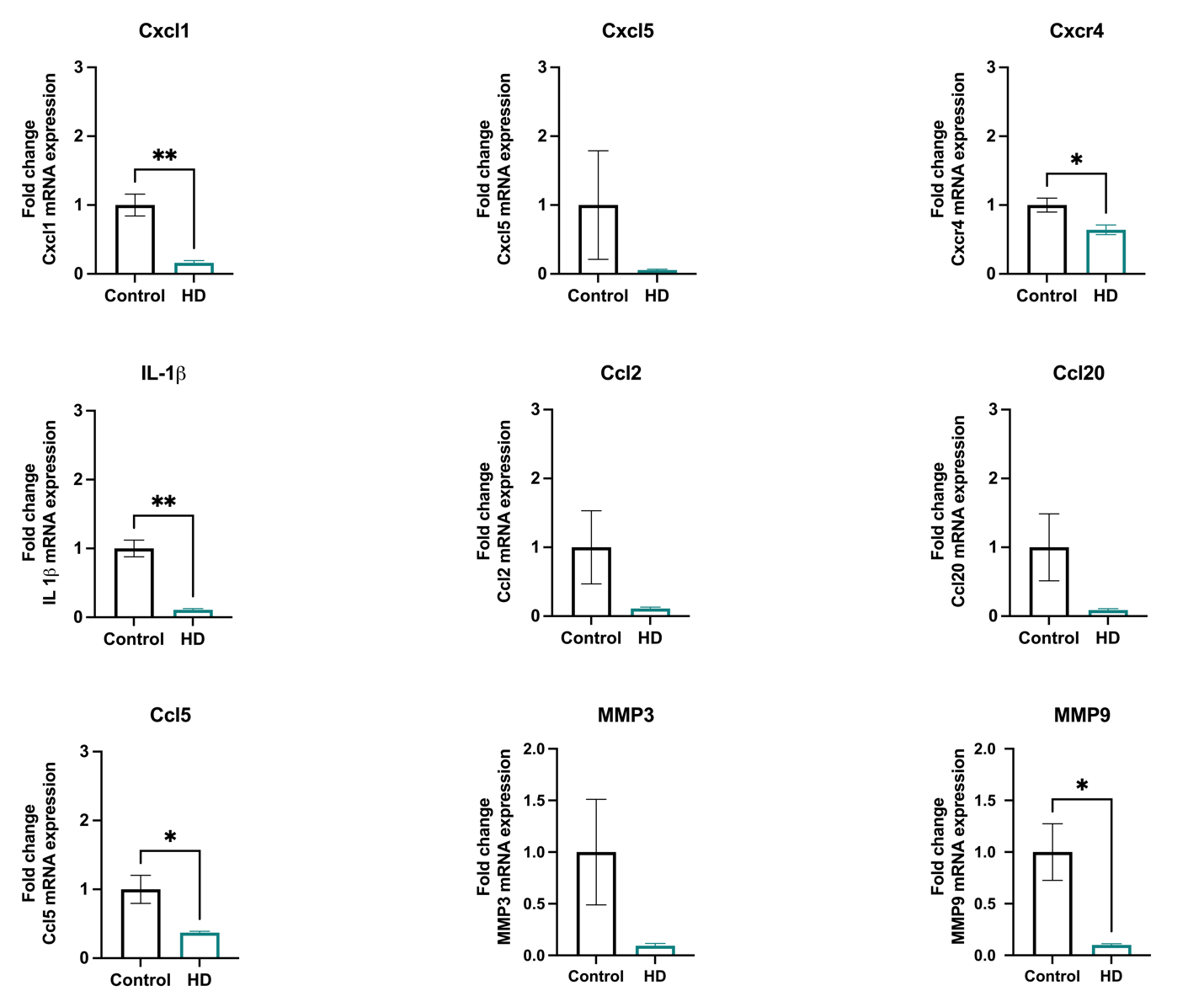


**Supplementary Figure S2. Quantification of hub gene mRNA expression via qPCR.** Gene expression of important StringDB PPI hub genes including inflammatory cytokines and matrix metalloproteinases were validated via qPCR. Cxcl1, Cxcr4, IL-1β, Ccl5, and MMP9 gene expression was significantly lower in the HD group in comparison to the control. Cxcl5, Ccl2, Ccl20, and MMP3 mRNA expression was non-significant between the groups, despite exhibiting a downward trend in the HD group than the control. Gene expression was normalized to the reference gene, Gusb. Bar plots are represented as mean with SEM. * p ≤ 0.05 and ** p ≤ 0.01.


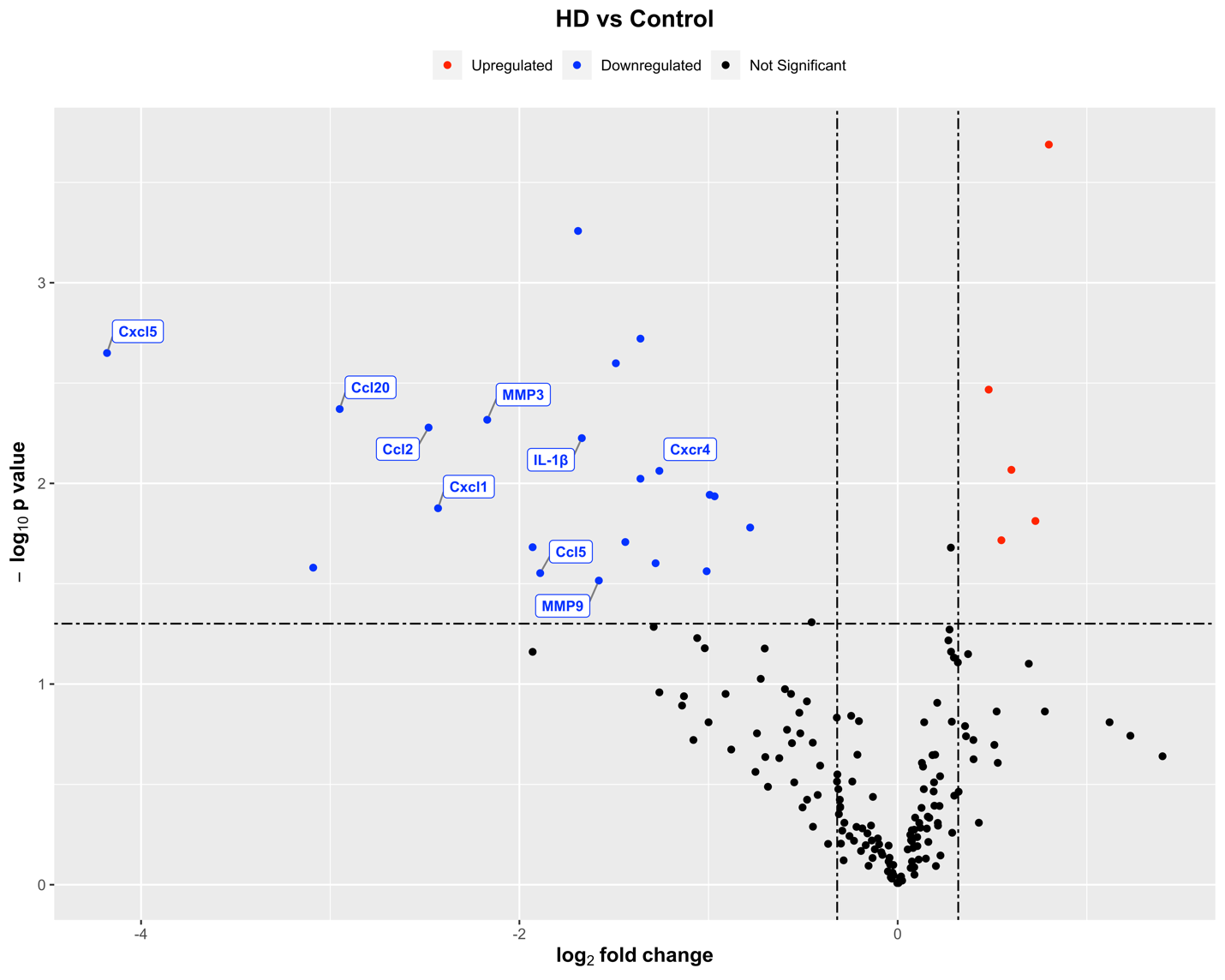


**Supplementary Figure S3. Downregulation of StringDB** **PPI hub genes.** Seven out of 10 hub genes identified from the StringDB PPI on the Cytoscape platform were inflammatory cytokines and chemokines. These hub genes (IL-1β, Cxcl1, Cxcl5, Cxcr4, Ccl2, Ccl20, and Ccl5) were significantly downregulated upon NanoString differential expression analysis. Amongst the top 10 hub genes, matrix metalloproteinases MMP3 and MMP9 were also identified to be significantly downregulated.


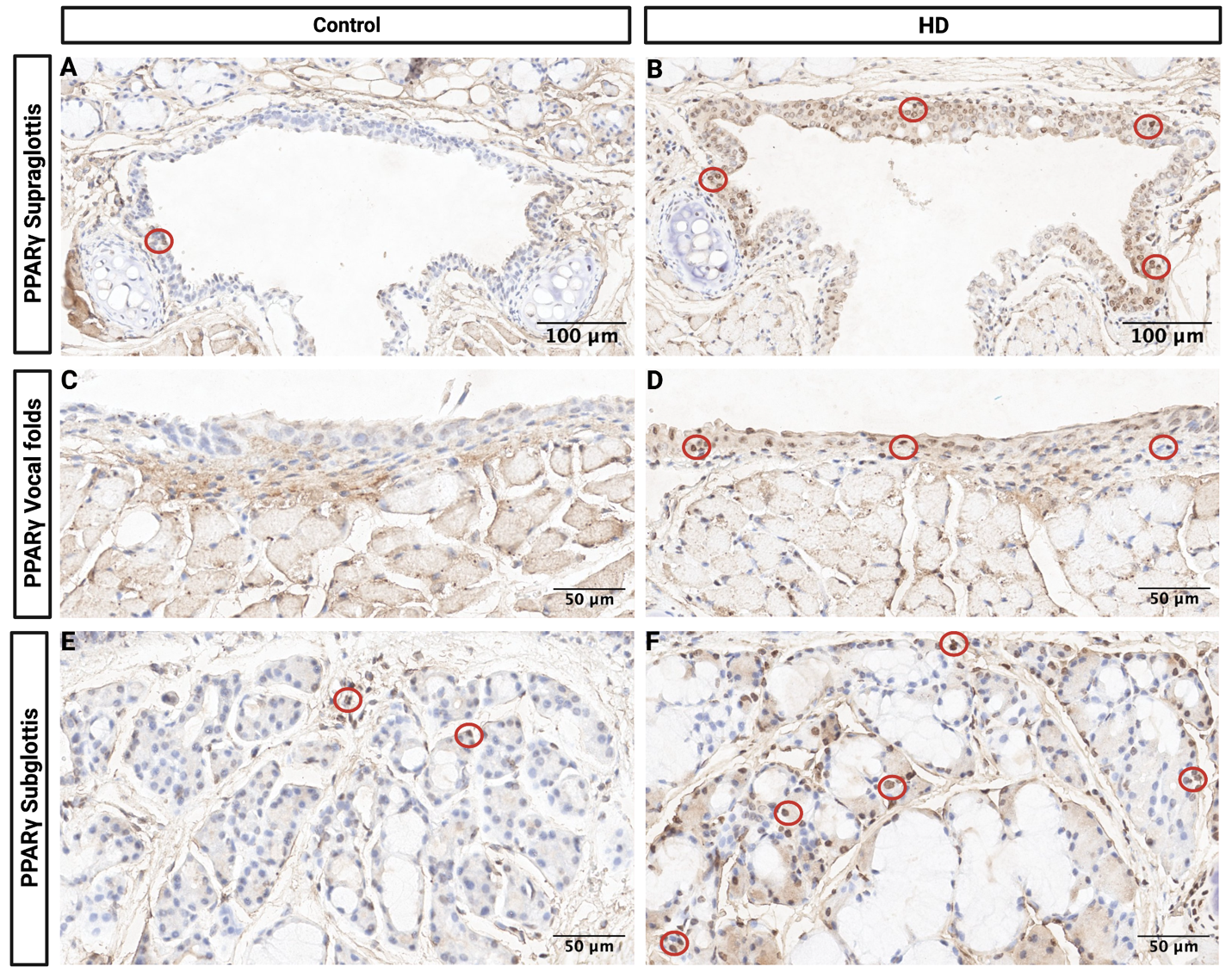


**Supplementary Figure S4. Immunohistochemical staining of** **PPARγ in the laryngeal mucosa.** HD CSE had no significant impact on the PPARγ levels in the laryngeal supraglottic (A, B), vocal fold (C, D), and subglottic (E, F) regions, although all the regions show a strong inclination towards increased PPARγ levels. Red circles indicate representative PPARγ labeled cells. Supraglottic images are at a magnification of 200x. Vocal fold and subglottic images are at a magnification of 400x.


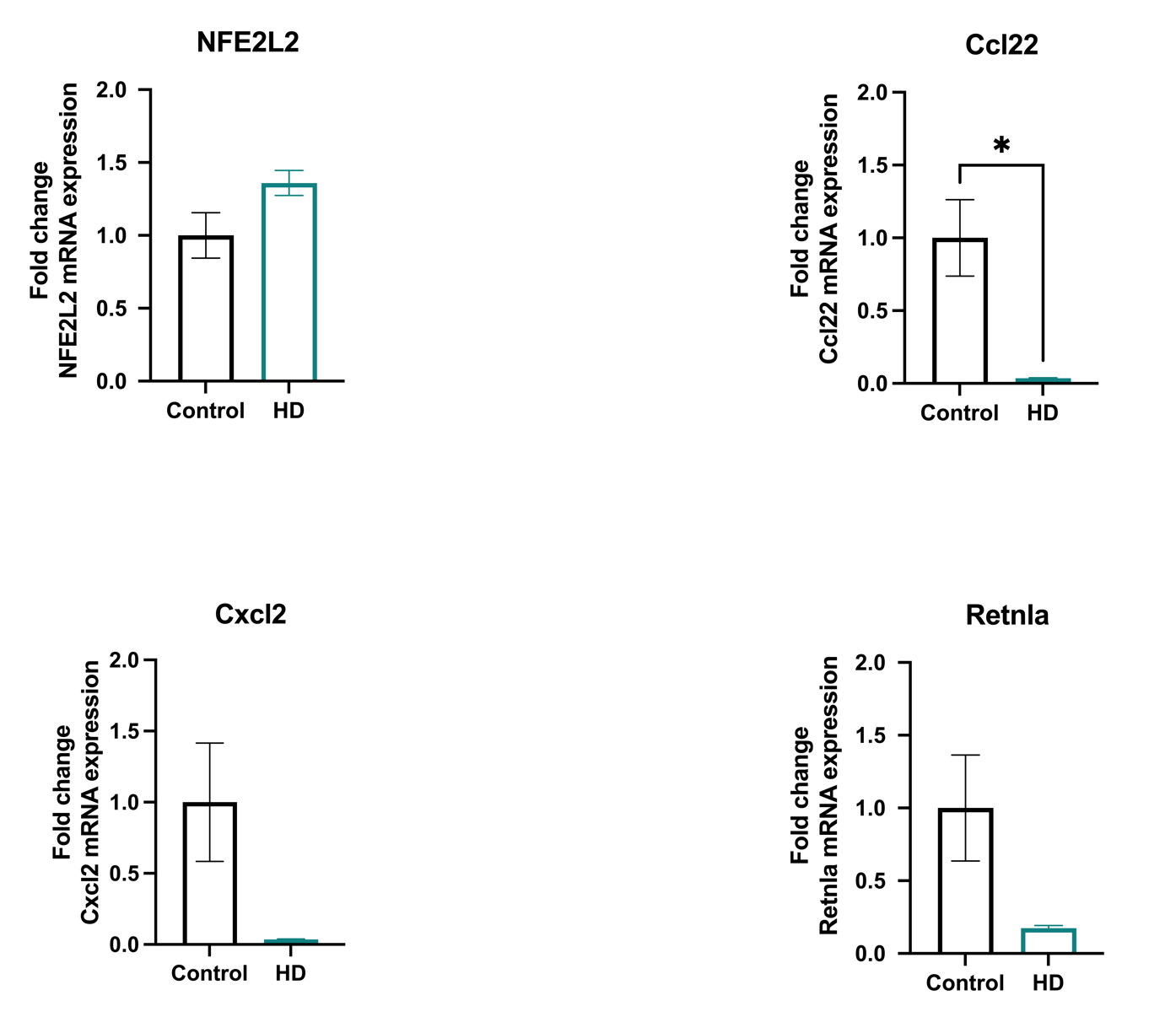


**Supplementary Figure S5. qPCR validation of PPARγ-regulated genes as prioritized by** **IPA.** NFE2L2, Ccl22, Cxcl2, and Retnla were identified as PPARγ regulated genes upon IPA. NFE2L2 was also identified as an upstream regulator by IPA. qPCR validation of NFE2L2 showed no major differences between both groups, despite having an upward expression trend in the HD group. Gene expression level of Ccl22 was significantly lesser in the HD group than control, whereas Cxcl2 and Retnla were non-significant, displaying a strong downward trend in gene expression upon HD CS exposures. Gene expression was normalized to the reference gene, Gusb. Bar plots are represented as mean with SEM. * p ≤ 0.05.

~~
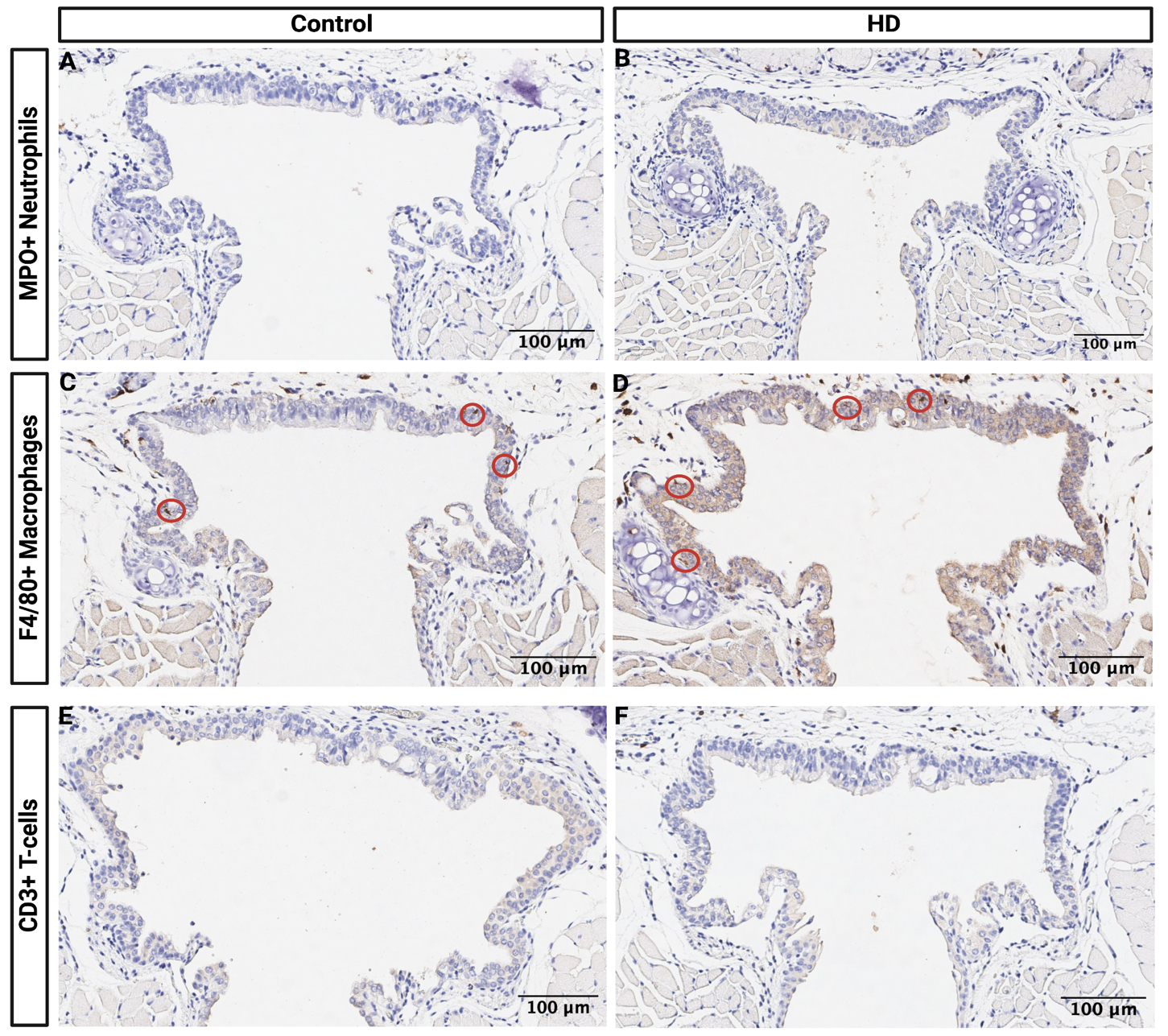
~~

**Supplementary Figure S6. Immunohistochemical staining of immune cells in the Supraglottis.** HD CSE had no impact on the MPO+ neutrophil (A, B), F4/80+macrophage (C, D), and CD3+T-cell (E, F) levels in the supraglottic regions of control and HD groups. Red circles indicate representative F4/80+macrophages. Supraglottic images are at a magnification of 200x.

**
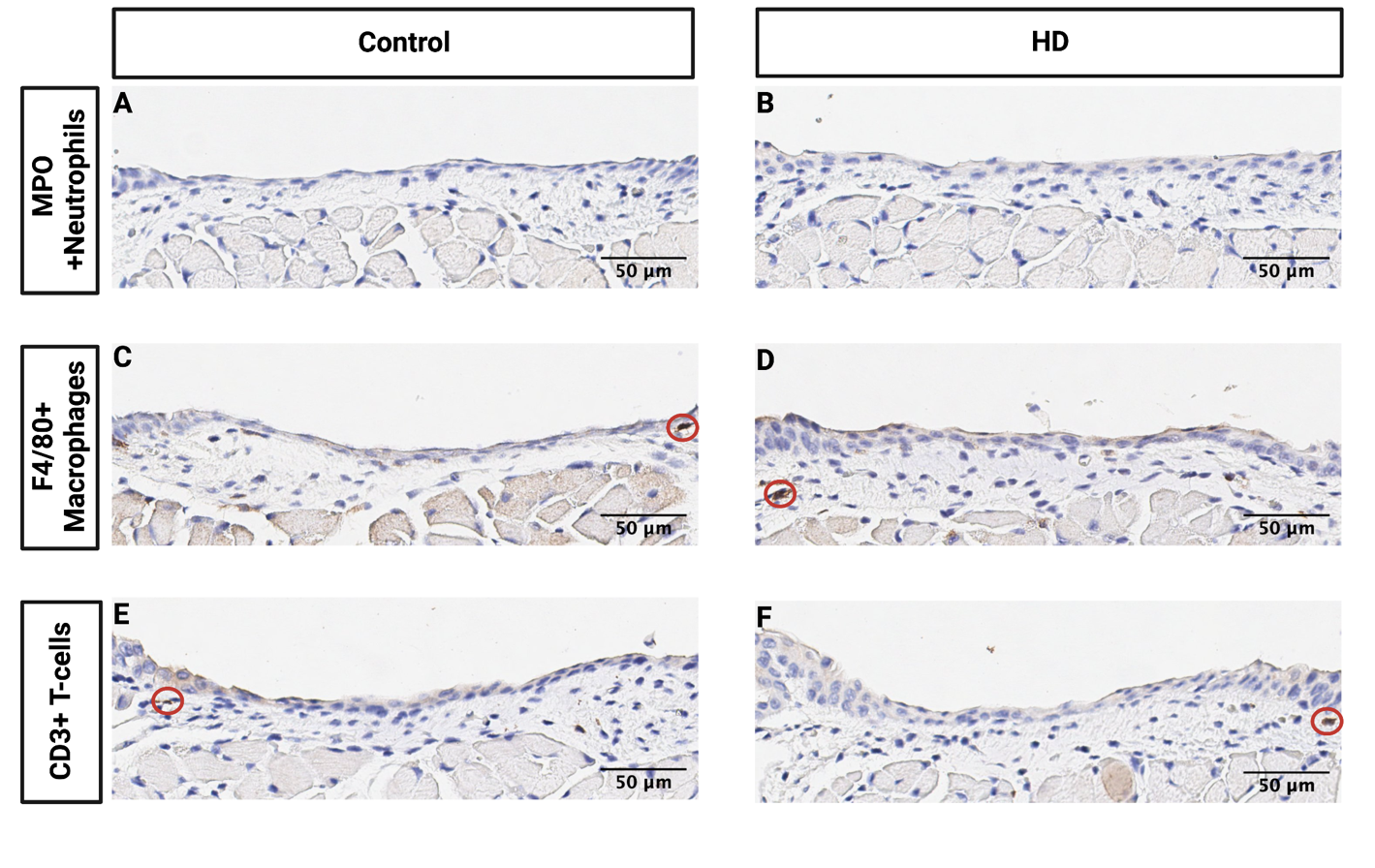
**

**Supplementary Figure S7. Immunohistochemical staining of immune cells in the vocal folds.** HD CSE had no impact on the MPO+ neutrophil (A, B), F4/80+macrophage (C, D), and CD3+T-cell (E, F) levels in the vocal fold regions of control and HD groups. Red circles indicate representative F4/80+macrophages or CD3+T-cells. Vocal fold images are at a magnification of 400x.

**
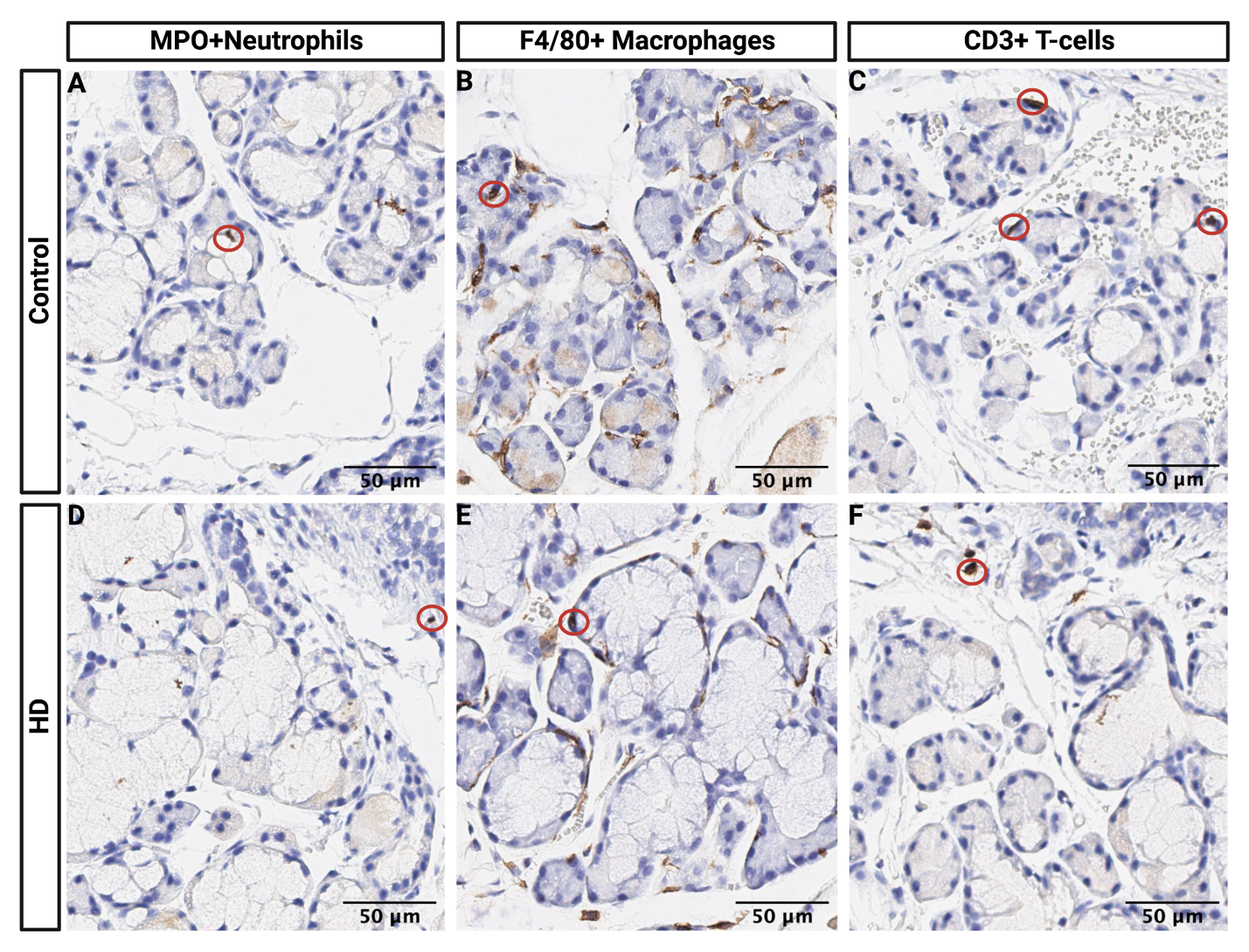
**

**Supplementary Figure S8. Immunohistochemical staining of immune cells in the Subglottis.** HD CSE had no impact on the MPO+ neutrophil (A, D) and F4/80+macrophage (B, E) levels in the subglottic regions of control and HD groups. Subglottic CD3+T-cell levels were significantly downregulated in the HD group than in the control (C, F). Red circles indicate representative MPO+ neutrophils or F4/80+macrophages or CD3+T-cells. Subglottic images are at a magnification of 400x.

~~
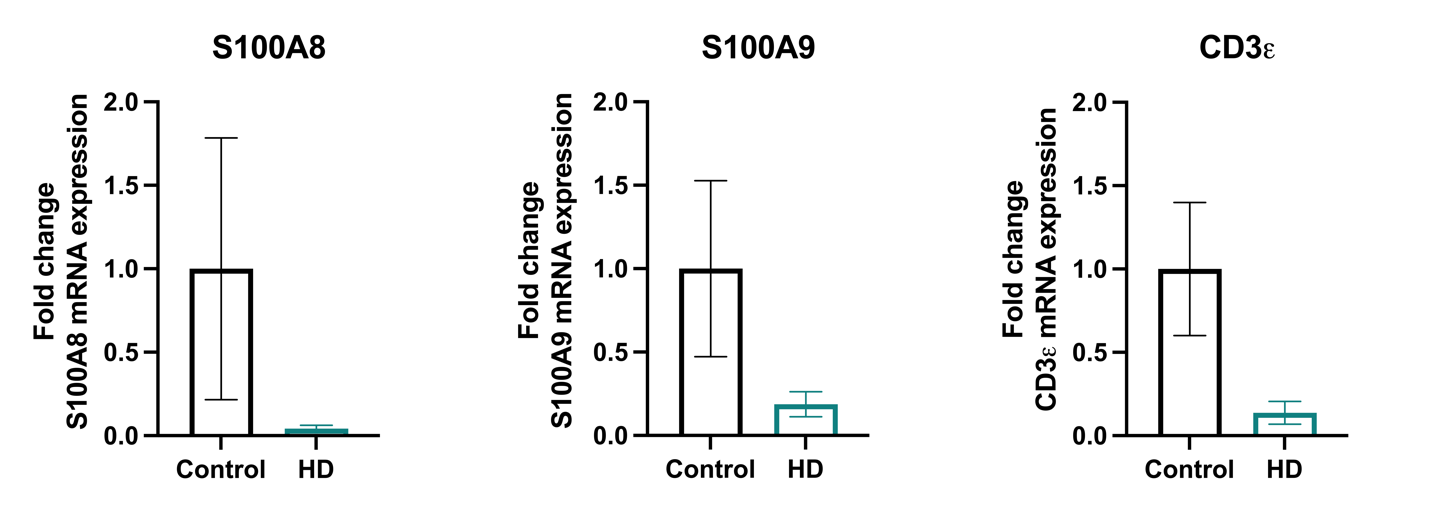
~~

**Supplementary Figure S9. Quantification of immune cells via qPCR.** Gene expression levels of neutrophil- and macrophage-specific markers like S100A8 and S100A9 were estimated via qPCR. In addition, expression levels of CD3-epsilon polypeptide (CD3ε) specific for all T-cell populations were also quantified via qPCR. mRNA expression levels for all three genes remained non-significant, despite exhibiting a strong tendency towards downward gene expression. Gene expression was normalized to the reference gene, Gusb. Bar plots are represented as mean with SEM.


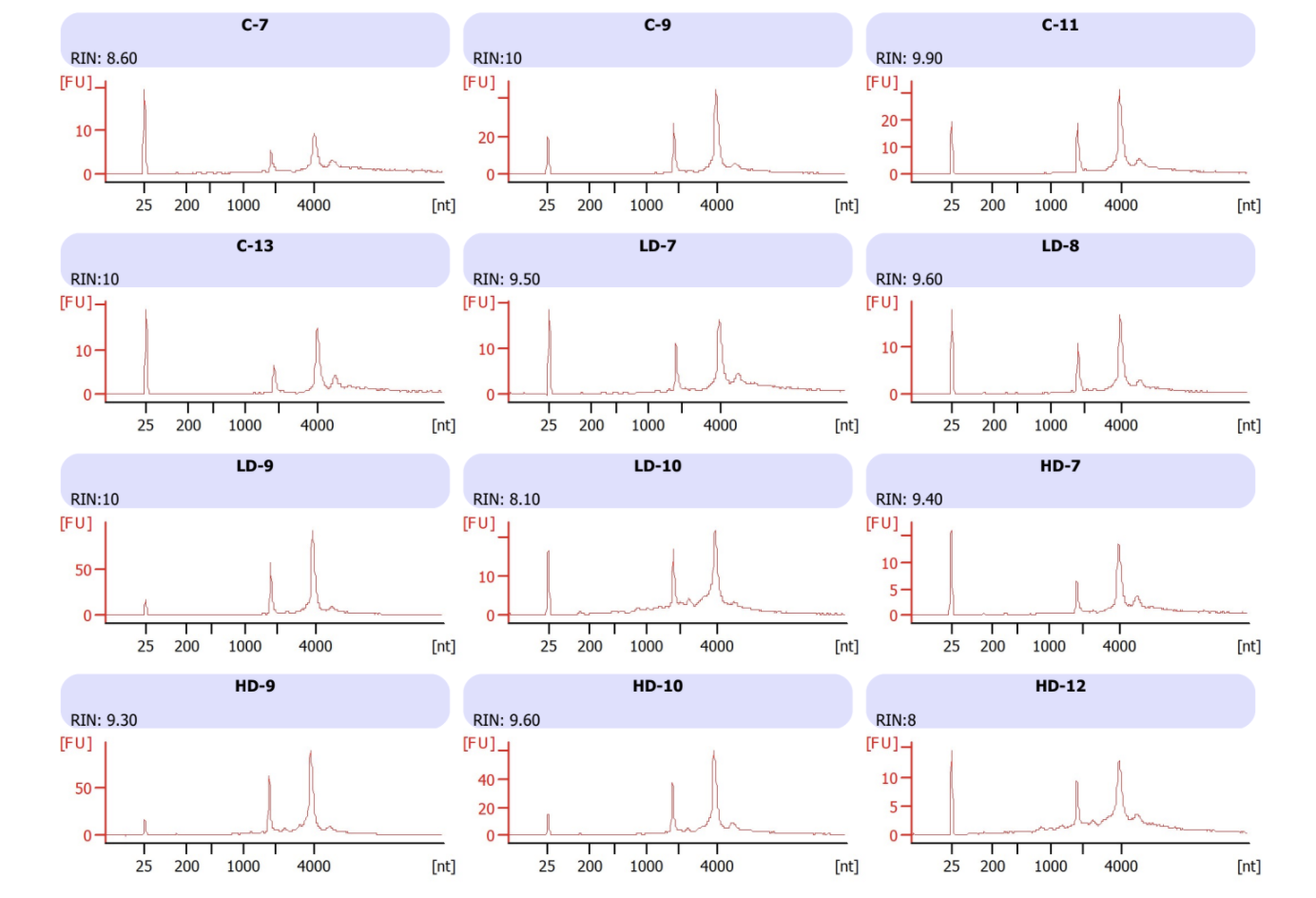


**Supplementary Figure S10. Assessment of RNA integrity numbers (RIN) for experimental samples.** Using Agilent Bioanalyzer 2100, RIN values were computed for the total mouse laryngeal RNA samples isolated from all the experimental groups, control, LD, and HD (n = 4 each). All samples had RIN > 8 and were selected for subsequent NanoString analysis. Control sample names: C-7, C-9, C-11, and C-13; LD group sample names: LD-7, LD-8, LD-9, and LD-10; HD group sample names: HD-7, HD-9, HD-10, and HD-12. All analyses were executed by Stanford Protein and Nucleic acid core facility (PAN).


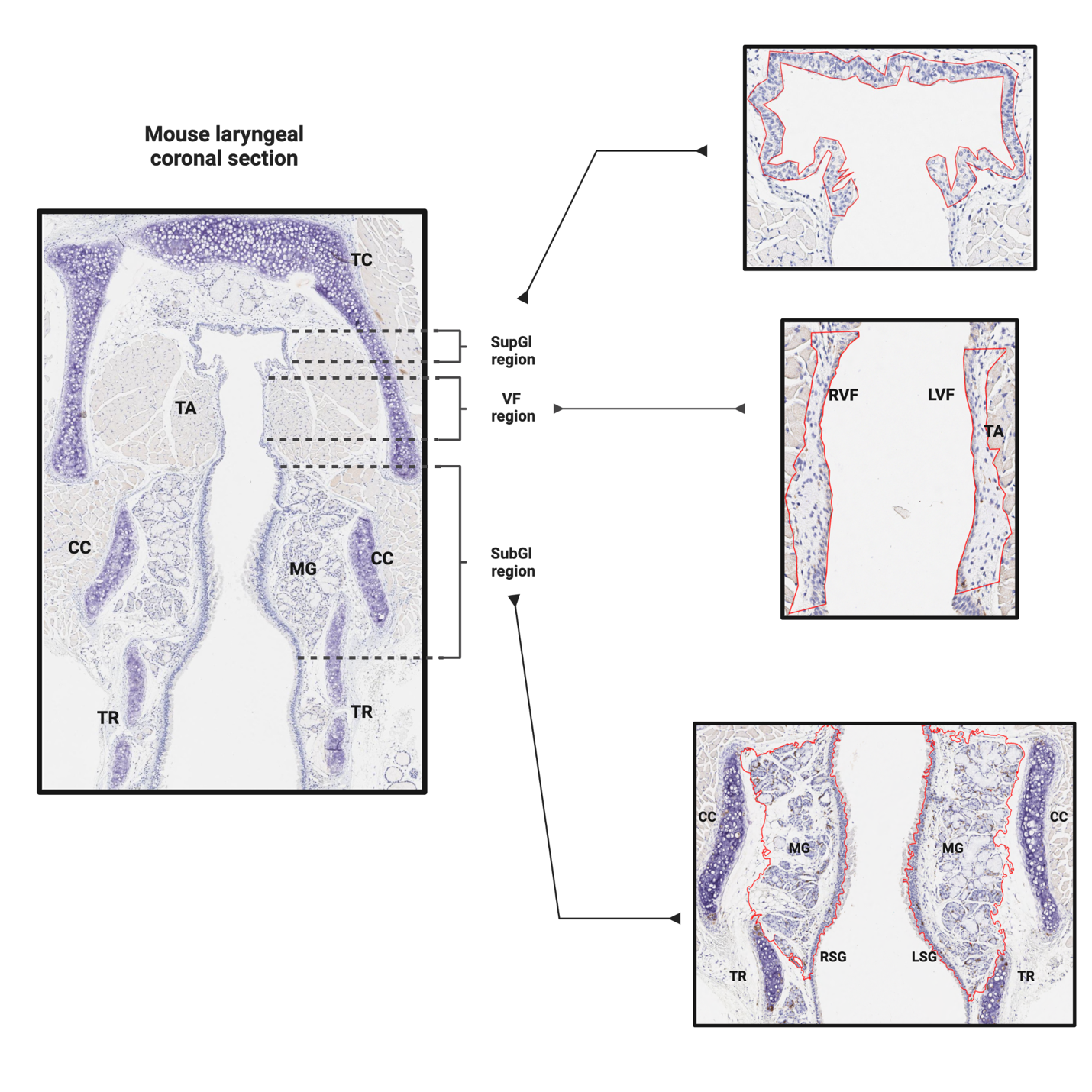


**Supplementary Figure S11. Anatomical landmarks for supraglottic, vocal folds, and subglottic regions in a murine laryngeal coronal section.** Within a coronal section of the mouse larynx, supraglottic (SupGl), vocal fold (VF), and subglottic (SubGl) regions can be viewed simultaneously and are well distinguished. VF regions are lined by stratified squamous epithelium with underlying layers of lamina propria and thyroarytenoid (TA) vocalis muscle. SupGl regions are lined by stratified squamous epithelium. SubGl regions are lined by pseudostratified ciliated columnar respiratory epithelium and have underlying mucosal glands (MG) flanked by cricoid cartilage (CC). For immunohistochemical assessment of immune cell infiltrates and PPARγ, the entire supraglottic region, and the mid-membranous regions on the right and left vocal fold (RVF/LVF) were traced. Regions starting from the lower end of VF regions until the first tracheal ring (TR) on right and left sides are the subglottic regions (RSG/LSG) and these were also traced to quantify these markers. All tracings were done in QuPath v 3.0 and are highlighted in red. TC: Thyroid cartilage
